# Integrative Prognostic Machine Learning Models in Mantle Cell Lymphoma

**DOI:** 10.1158/2767-9764.CRC-23-0083

**Published:** 2023-08-02

**Authors:** Holly A. Hill, Preetesh Jain, Chi Young Ok, Koji Sasaki, Han Chen, Michael L. Wang, Ken Chen

**Affiliations:** 1 Department of Bioinformatics and Computational Biology, Division of Quantitative Sciences, The University of Texas MD Anderson Cancer Center, Houston, Texas.; 2 Department of Lymphoma and Myeloma, Division of Cancer Medicine, The University of Texas MD Anderson Cancer Center, Houston, Texas.; 3 Department of Epidemiology, Human Genetics and Environmental Sciences, The University of Texas Health Science Center at Houston School of Public Health, Houston, Texas.; 4 Department of Hematopathology, Division of Pathology-Lab Medicine, The University of Texas MD Anderson Cancer Center, Houston, Texas.; 5 Department of Leukemia, Division of Cancer Medicine, The University of Texas MD Anderson Cancer Center, Houston, Texas.; 6 Center for Precision Health, School of Biomedical Informatics, The University of Texas Health Science Center at Houston, Houston, Texas.

## Abstract

Patients with mantle cell lymphoma (MCL), an incurable B-cell malignancy, benefit from accurate pretreatment disease stratification. We curated an extensive database of 862 patients diagnosed between 2014 and 2022. A machine learning (ML) gradient-boosted model incorporated baseline features from clinicopathologic, cytogenetic, and genomic data with high predictive power discriminating between patients with indolent or responsive MCL and those with aggressive disease (AUC ROC = 0.83). In addition, we utilized the gradient-boosted framework as a robust feature selection method for multivariate logistic and survival modeling. The best ML models incorporated features from clinical and genomic data types highlighting the need for correlative molecular studies in precision oncology. As proof of concept, we launched our most accurate and practical models using an application interface, which has potential for clinical implementation. We designated the 20-feature ML model–based index the “integrative MIPI” or iMIPI and a similar 10-feature ML index the “integrative simplified MIPI” or iMIPI-s. The top 10 baseline prognostic features represented in the iMIPI-s are: lactase dehydrogenase (LDH), Ki-67%, platelet count, bone marrow involvement percentage, hemoglobin levels, the total number of observed somatic mutations, *TP53* mutational status, Eastern Cooperative Oncology Group performance level, beta-2 microglobulin, and morphology. Our findings emphasize that prognostic applications and indices should include molecular features, especially *TP53* mutational status. This work demonstrates the clinical utility of complex ML models and provides further evidence for existing prognostic markers in MCL.

**Significance:**

Our model is the first to integrate a dynamic algorithm with multiple clinical and molecular features, allowing for accurate predictions of MCL disease outcomes in a large patient cohort.

## Introduction

Mantle cell lymphoma (MCL) is a rare, incurable B-cell malignancy with heterogeneous clinical outcomes and molecular pathogeneses. Most patients relapse after treatment and have disease progression, while others have an indolent form of the disease or respond exceptionally to frontline therapy and achieve durable remissions (1). Currently, targeted and cellular therapies are being explored as frontline treatment alternatives to high-dose chemotherapy (2). Identifying predictive biomarkers for integration in prognostic models is important for stratifying patients and making therapeutic decisions.

Multiple studies have identified prognostic baseline clinicopathologic, genomic, and cytogenetic characteristics and biomarkers of MCL (3–11). In the Nordic MCL2 and MCL3 cohorts, *TP53* mutations were significant prognostic factors for overall survival (OS) and time to relapse. Furthermore, patients with *TP53* mutations did not respond well to intensive frontline chemoimmunotherapy (4). Aggressive histology has also been highlighted as a relevant prognostic feature. Blastoid or pleomorphic histology is associated with shorter OS and progression-free survival (PFS; ref. 12). In addition, a complex karyotype, defined by three or more cytogenetic aberrations, has also been linked to poor outcomes (13).

Advances in targeted therapies (2, 14) and molecular profiling (15) add an advantageous dimension to clinical risk stratification and improve survival outcomes. Indices such as the mantle cell lymphoma international prognostic index (MIPI), and subsequently, the biological MIPI (MIPI-b) and simplified MIPI (MIPI-s) were constructed using data from patients treated with intensive frontline chemoimmunotherapies (12, 16). These indices incorporate limited clinical features and do not include molecular or histopathologic data. A new prognostic score was proposed that integrates seven clinical features with a clustering algorithm, the engineered MIPI (eMIPI; ref. 17). The eMIPI improves upon the limited discernment of the restricted feature MIPIs but does not include data from cytogenetic or next-generation sequencing (NGS) studies. Prognostic MCL studies implementing molecular features have demonstrated higher accuracy than clinical markers alone in predicting survival (18).

Machine learning (ML) methods promise to advance precision oncology for MCL and other malignancies. ML has been employed in lymphoma and leukemia studies to build prognostic models (19, 20), diagnose disease (21), integrate radiomics to identify central nervous system lymphoma (22, 23), and classify cell states of the tumor microenvironment (24, 25). ML models integrating baseline molecular, pathologic, and clinical data in oncology outperform traditional statistical models, especially in datasets with several predictive features (26, 27). However, many ML techniques require large datasets that are difficult to procure in rare malignancies. Studies of rare cancers typically involve small patient cohorts and multiple features of interest. Harmonizing large combined multi-institutional datasets may be a partial solution to this problem in addition to focusing on informed feature selection and engineering.

Decision trees capture the nonlinear relationship between a classification label (or numerical value in regression) and multiple features. Many features relevant to cancer prognosis are not linearly related to outcomes. For example, a very high or low body mass index (BMI) could lead to worse survival outcomes in cancer, and specifically lymphoma, for diverse reasons (28, 29). High white blood cell (WBC) counts are usually associated with high disease burden and advanced disease in many hematologic malignancies, but high WBC counts in leukemic non-nodal MCL are not associated with inferior outcomes (1).

Large patient databases may have large amounts of missing clinical and genomic data. Gradient-boosted ensemble tree models are well suited for making predictions in cohorts with many categorical predictors and large amounts of missing data. In particular, the extreme gradient boosted (XGBoost) library is optimized for missing data utilizing a sparsity aware split-finding algorithm and does not require imputation by other statistical methods (30).

Boosted decision-tree classifiers work well with overlapping or collinear features. For two perfectly correlated features (A and B), each independent tree in an ensemble chooses the best variable, learned by the algorithm, and places all of the importance on A or B, but not both (as is the case in random forest models; refs. 30, 31). Unlike a random forest model, XGBoost initially “prunes” its trees using a “similarity score” before it starts iterative modeling. Although more computationally expensive, this prevents the model from overfitting and choosing homogenous trees when similar samples or observations represent the majority of a dataset. Moreover, the XGBoost algorithm improves when it fails to predict rare outcomes by giving more weight to that outcome (or class) in following iterations improving its performance in datasets with class imbalance compared with the random forest algorithm (30).

XGBoost and other gradient-boosted tree ensemble algorithms aggregate multiple weak classifiers into stronger classifiers by iteratively utilizing a series of regression or classification trees. In a complex system such as the pathophysiology of cancer, which comprises multiple heterogeneous features with varying levels of predictive strength, nonlinear models can provide insight into targetable biomarkers or modifiable exposures. Gradient-boosted trees can also be effectively used for feature selection and unsupervised classification for integration into additional statistical models (32, 33).

Determining feature importance is unique to ML methods and differs from traditional hypothesis testing using inferential statistics (34). Random forests and gradient-boosted methods use this variable improvement or importance score for each predictor and average it for all the trees in the ensemble (35). In addition, Shapley (SHAP) additive values are used in complex nonlinear models, such as XGBoost, to explain the impact of several variables on the dependent variable(s) and are based on cooperative game theory, where many participants contribute to the outcome (36, 37).

As in traditional logistic models, metrics such as the area under the ROC curve (AUC ROC) are used to assess the performance of an ML model (38). In addition, sensitivity, specificity, likelihood ratios, and predictive values are used to describe the clinical utility of a model to discriminate between true or false positives and negatives (39, 40). The capability to predict the clinical course is important. However, high sensitivity and positive predictive value (PPV) are imperative for identifying patients who require aggressive therapy at baseline (41). Conversely, patients with predicted indolent disease could be identified to receive less aggressive frontline treatment. Multiple chemotherapy-free induction and frontline strategies are currently being trialed in MCL (2, 14, 42). Balancing sensitivity and specificity is reflected in the AUC ROC metric.

In this study, we incorporated baseline clinicopathologic, molecular, and cytogenetic features in ML models to predict outcomes in a large cohort of patients with MCL. This integrative approach embraces previous research that associated patient outcomes with numerous biomarkers and other diagnostic measures.

## Materials and Methods

### Database Curation and Feature Engineering

We retrospectively curated an extensive database of patients with MCL (*n* = 862) treated at our institution (University of Texas MD Anderson Cancer Center, Houston, TX) between January 1, 2014 to June, 30 2022.

Patient data were extracted from electronic medical records (EMR) using approved methods from our Institutional Review Boards (IRB). The samples used for genetic sequencing were collected according to our approved IRB laboratory protocol for tissue banking. Sample and data collection were done with IRB-approved written informed consent and in accordance with ethical principles contained in the Belmont Report and Declaration of Helsinki.

**FIGURE 1:**
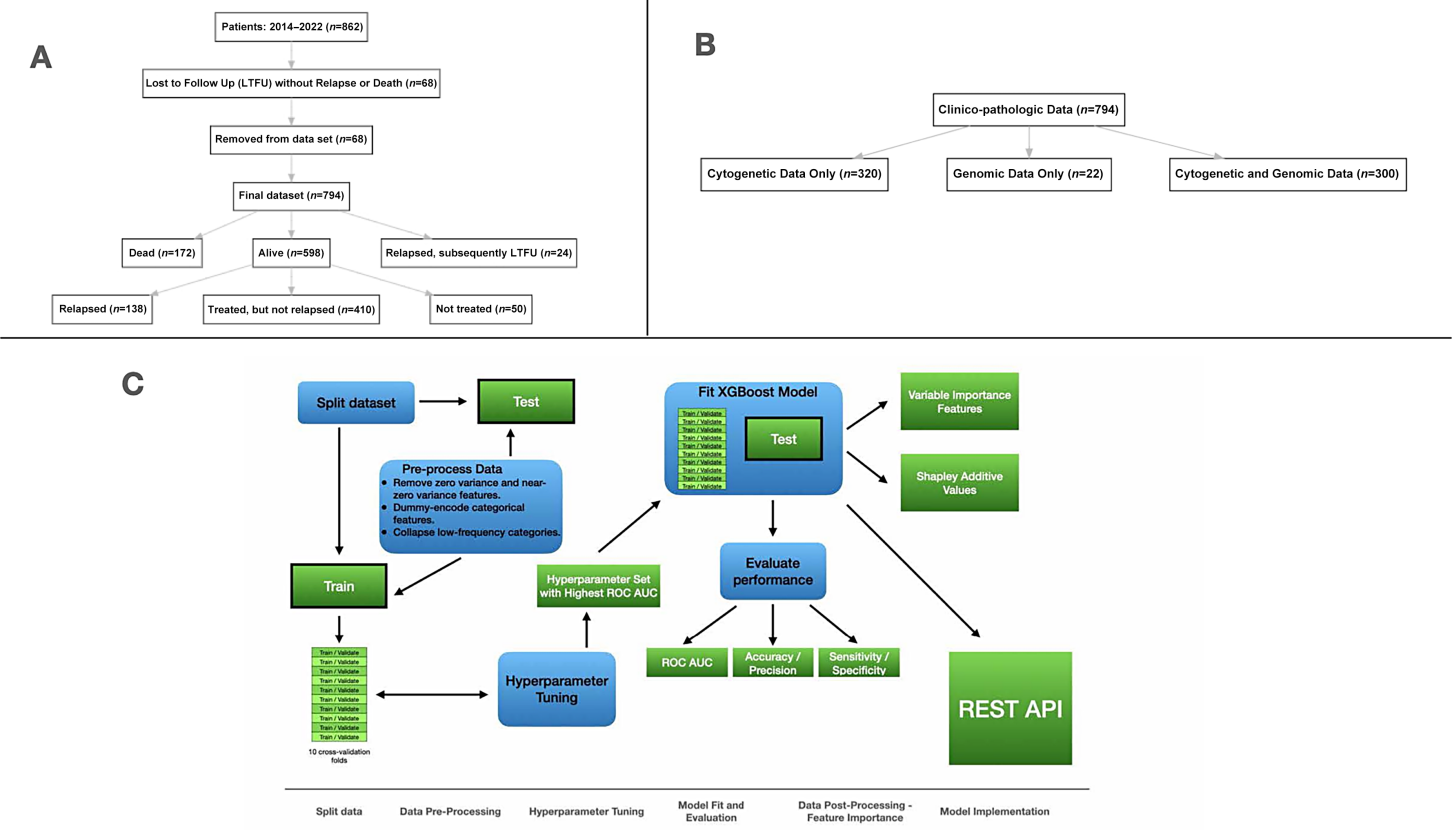
Selection process and workflow of prognostic models. **A,** Flowchart depicting MCL patient selection process for inclusion in models. **B,** Flowchart showing data availability for patient cohort (*n* = 794). All patients had clinicopathologic data. Most patients (*n* = 642) had cytogenetic and/or genomic data. **C,** Workflow of ML (XGBoost modeling). The dataset containing all 794 patients was split into a training/validation set and a test set. The test set was held from all initial preprocessing and hyperparameter tuning to avoid data leakage. Data preprocessing included removing zero and NZV features, dummy encoding categorical features, and collapsing low-frequency categorical variables into an “other” category. The training set was again split into 10 cross-fold validation sets where hyperparameters for the XGBoost model were tuned. The hyperparameter set with the highest mean ROC AUC was chosen for the final fit on the test set, and performance was evaluated to check the model's fit. Variable importance was visualized with a VIP and SHAP additive values. The fitted XGBoost model was launched using a REST API, demonstrating clinical utility.

Patients who were seen only once for a consultation or second opinion were excluded from the cohort. Patients who did not have an outcome of interest (death, relapse, or disease progression) were followed for a minimum of 6 months. Outcome information in the patient database was updated in January 2023. Data were extracted from 862 patients: 68 patients were removed who were lost to follow-up (LTFU) and did not have relapse, disease progression, or death (status unknown); 794 patients were included in the final dataset for the model (Fig. 1A).

We extracted baseline clinical information, including multiple variables ranging from clinical laboratory data to patient-reported exposures. Clinical demographics included age at diagnosis, race/ethnicity, and biological sex. Other clinical data such as weight and height (BMI), and smoking status and alcohol use (drinks per week) were collected. Treatment data were collected, but not used in model inference due to ongoing clinical trials and heterogeneity of length and types of treatment (Supplementary Table S1).

In addition, we collected data from pathology reports, including morphology and proliferation indices, and cytogenetic data from FISH, comparative genomic hybridization, and conventional karyotyping. Genetic data, including somatic and germline mutations, were curated from previously published studies by our group (2, 14, 7) and targeted sequencing panels implemented in the clinical setting (43). Full information pertaining to sample processing, sequencing, and bioinformatic analyses of genomic and cytogenetic data are reported in our Supplementary Materials and Methods. Features from clinicopathologic measures, cytogenetics, and DNA sequencing were collected, and certain composite features were generated. Outcome variables were determined from clinical endpoints such as remission, relapse, disease progression, and death. Patients who were lost to follow-up were identified, and the last follow-up date was recorded.

Features with no observations or variance (e.g., genes in gene panels that yielded no patient mutations) were removed. Selected features were engineered by creating composite variables based on prior research, such as an interaction term when *TP53* and *ATM* mutations occurred together (6, 44).

A complex karyotype is a standard feature defined by contributing pathologists from the clinical record, which refers to three or more unrelated cytogenetic abnormalities (13). In addition, an “abnormal karyotype” variable was created for patients with three or more cytogenetic abnormalities, whether related or unrelated, and not coded as “complex” by contributing pathologists. Although these two features are colinear, the XGBoost model accommodates codependent features by choosing one of the overlapping variables to be used in a decision tree (31).

Several chromosomal aberrations had few recorded instances, resulting in near-zero variance (NZV); abnormalities on the same chromosome were combined to represent these phenomena. For example, all translocations involving chromosome 1 were recorded as “chromosome 1 other”; if the translocation also involved chromosome 9, [i.e., t(1:9)], then an observation of “chromosome 9 other” would also be recorded. This allows a less granular molecular abnormality to be represented as a feature in the model, which is not specific to any gene or chromosomal region. A complete list of single-variable, engineered features and variables removed owing to low variance is shown in Supplementary Table S2.

Patients who relapsed or progressed after frontline treatment or died without treatment due to an aggressive onset of MCL were classified as having “aggressive” MCL. Patients who did not require treatment or remained in remission after frontline treatment were labeled as having “indolent/responsive” MCL. For survival models, PFS and OS were calculated for eligible patients.

Of the patients included in the models, 794 had clinical data, 622 had cytogenetic data, and 320 had genomic data (Fig. 1B). There were various levels of missing data among features (Supplementary Table S3).

### ML Models

After data extraction and feature engineering, 129 predictive features were incorporated into an extreme gradient-boosted ensemble ML model (XGBoost). A set seed randomly split the dataset into a test and training set (75/25). The training set was divided into 10 folds (75/25) for cross-validation (validation set). To avoid class imbalance, the training, validation, and testing sets were split using the stratification of the classification variable. The training set contained 595 patients, and the test set comprised 199 patients.

Hyperparameters are internal configurations or settings of an ML model that optimize performance. The hyperparameters for all models were tuned using a grid-based, space-filling (Latin hypercube) technique, and resampled 10-fold cross-validation (validation set). Hyperparameters were tuned by setting the number of trees and leaving the following open for tuning: tree depth, loss reduction, sample size, and learning rate. Early stopping was employed to avoid overfitting and decrease the computational time. A Latin hypercube of size 50 was used for the grid-based search on the resampled folds. Tuning was performed using the ROC AUC metric. The hyperparameter fit with the highest ROC AUC, averaged over the 10 folds, was chosen for the final model and incorporated into the model workflow.

Data were preprocessed by creating dummy variables for all nominal predictors, including somatic gene mutations. Mutations were coded as “yes” = mutated, “no” = not mutated, or left missing to indicate that the mutation was not interrogated (in the case of the targeted panels). Features with less than 1% representation were collapsed into an “other” category. All nominal features with NZV were removed.

The trained model was fit on the test set. Metrics including accuracy, ROC AUC, precision, sensitivity, and specificity were collected. Variable importance plots (VIP) and SHAP additive values were generated. The workflow of the XGBoost models is illustrated in Fig. 1C.

Four models were initially generated for performance comparison: clinical data only, clinical plus NGS data, clinical plus cytogenetic data, and a combined model with all data (“all features model”). Single variable–type models were also constructed and evaluated.

Implementing the robust feature selection ability of XGBoost, two parsimonious models were constructed by averaging variable importance over the 10 cross-validation folds from the “all features” model training set (Supplementary Fig. S2). The top 10 and 20 variables were selected for implementation in the parsimonious models, with fewer features demonstrating clinical utility and scalability. Categorical features were transformed into dummy variables resulting in 12 and 24 individual binary features for the respective models. We applied the same workflow depicted in Fig. 1C to construct the reduced model.

The top 10 and 20 VIPs and SHAP values from all XGBoost model test sets were summarized and visualized using the R libraries: SHAP for XGBoost (45) and VIP (46).

To assess the ability and robustness of XGBoost's split-finding algorithm for dealing with missing data, we constructed a “complete cytogenetic case” model where we pared the database down to the 616 patients who had complete cytogenetic data. Of these patients, 299 had some mutational data available.

### Comparative Models

#### Random Forest Model

To demonstrate the improved accuracy and utility of the XGBoost models, a comparative random forest model was constructed using the top 20 features identified by variable importance (analogous to the parsimonious model). Data preprocessing and data splitting was identical to the XGBoost models with the exception of imputation as gradient boosted models handle missing values directly. Imputation of missing values was done by bagged imputation using all predictive variables. Hyperparameters including mtry and the minimum n were tuned using a grid-based approach with 2,000 trees.

#### Generalized Linear Model

A multivariate logistic model was generated to compare the impact of the important features on a traditional statistical model. The top 20 features identified by variable importance from the all-feature XGBoost model were used to construct a generalized linear model (GLM) with logistic regression to predict the same binary classification (aggressive vs. indolent/responsive). Similar to the XGBoost model, the data were stratified by the outcome variable and split into training and test sets, with the training set being split into folds for cross-validation.

Numerical variables were examined for multicollinearity (Supplementary Fig. S4). Data preprocessing included the imputation of all predictor variables using the k-nearest neighbor (KNN) technique. Nominal variables with low variance were collapsed into an “other” category, and dummy variables were created from all nominal predictors to avoid ordinal representation in the model. Metrics were collected, and variable coefficients, SEs, and *P* values for the GLM were extracted.

#### Survival Models

In addition to the GLM, Cox proportional hazards (PH) models were constructed from the top 10 and 20 identified features from XGBoost. We chose endpoints for two models using OS: time from diagnosis to death, and PFS: time from treatment to relapse, disease progression, or death.

All 794 patients were included in the OS model. Of the entire cohort, 718 patients received treatment and were included in the PFS dataset. Data preprocessing for the Cox PH models consisted of imputing missing predictor data using KNN imputation, normalizing all numeric predictors, and creating an “other” category for the categorical variable with multiple levels (morphology) where groups had less than 1% representation. Patients were considered censored if LTFU occurred before the event: death, relapse or progression for PFS, and death for OS.

Utilizing the top features identified in the full XGBoost model, we modeled OS and PFS with Kaplan–Meier curves. Differences in survival between/among groups were calculated with the log-rank test. We used the R libraries: survminer (47), survival, and ggsurvfit.

#### Comparison with Existing MCL Indexes

A model comparison was made with the existing MIPI and MIPI-b by constructing the index variables from the extracted data. The MIPI incorporated age at diagnosis, lactase dehydrogenase (LDH)/upper limit of laboratory normal (ULN), Eastern Cooperative Oncology Group (ECOG) performance status, and WBC count. The same variables plus Ki-67% were used to build the MIPIb. Univariate logistic models were created by predicting the outcome variable using the MIPI and MIPIb without similarly adjusting for covariates in the multivariate model. The ROC AUC and accuracy were calculated.

### Data and Code Availability

All model and visualization code are available at https://github.com/HAHill/machine_learning_MCL. The deidentified datasets are available upon request. Information on our representational state transfer application programming interface (REST API) can be found in our GitHub repository. Please note that the REST API prototype cannot be used in clinical practice and is proof of concept only; it has not been approved by the FDA.

## Results

### Patient Characteristics

Baseline patient characteristics including selected demographic, clinicopathologic, genomic, and cytogenetic variables are summarized in Table 1. The training and the test set had comparable characteristics. Characteristics between classified groups reflected previously reported profiles of patients with MCL (4, 14, 48).

**TABLE 1:** Characteristics of patients with MCL (*n* = 794)

	All (*n* = 794)	Indolent/ Responsive (*n* = 462)	Aggressive (*n* = 332)	Training set (*n* = 595)	Test set (*n* = 199)
Characteristics					
Age at diagnosis, median (range), years	63.3 (27–93.5)	61.9 (27–84)	64.5 (31.9–93.5)	63.4 (27–88.2)	62.15 (31.9–93.54)
Male, *n*, %	587, 73.9	334, 72.3	253, 76.2	444, 74.6	143, 71.9
Race or ethnicity, *n*, %					
White	703, 88.5	414, 89.6	289, 87	525, 88.2	178, 89.4
Black	15, 1.9	8, 1.7	7, 2.1	11, 1.8	4, 2.0
Hispanic	52, 6.5	28, 6.1	24, 7.2	42, 7.1	10, 5.0
Asian	10, 1.3	6, 1.3	4, 1.2	8, 1.3	2, 1.0
Other	14, 1.8	6, 1.3	8, 2.4	9, 1.5	5, 2.5
Ki-67 median, (range) %	30 (0–100)	23 (0–95)	40 (0–100)	30 (0–100)	30 (0–100)
Bone marrow involved, median, (range) %	10 (0–100)	8 (0–95)	20 (0–100)	10 (0–100)	15 (0–100)
Lactate dehydrogenase (ULN = 255)	258 (105–5,803)	232.0 (105–915)	399.5 (125–5,803)	249.5 (110–5,803)	300.5 (105–2,247)
Platelets, median, (range)	185 (6–796)	191 (19–698)	164 (6–796)	185.0 (6–796)	183 (19–698)
Hemoglobin, median, (range)	13.2 (1.8–27.1)	13.6 (1.8–18)	12.4 (4.7–27.1)	13.3 (1.8–27.1)	13.2 (5.9–17.4)
White blood cell count, median, (range)	7,200 (1,000–341,700)	6,900 (1,500–200,000)	7,600 (1,000–341,700)	7,100 (1,000–328,000)	7,300 (1,280–341,700)
Beta-2 microglobulin, median, (range)	2.8 (0.9–22.5)	2.5 (0.9–2.5)	3.3 (1.2–19.2)	2.8 (0.9–22.5)	2.8 (1.3–13)
BMI, median (range)	27.8 (16.5–66.5)	28.1 (16.5–66.5)	27.4 (17.8–48.4)	28.11 (16.5–66.5)	26.58 (19–44.9)
B-symptoms, *n*, %	162, 20.4	67, 14.5	95, 28.6	113, 19.0	49, 24.6
Smoking status, *n*, %					
Current	52, 6.6	27, 5.8	25, 7.5	39, 6.6	13, 6.5
Former	262, 33.4	135, 29.2	127, 38.3	201, 33.8	61, 30.7
Never	459, 58.5	291, 63.0	168, 50.6	339, 57.0	120, 60.3
Mutations, *n*, %					
*ATM*	114, 14.4, 35.4^a^	86, 18.6	28, 8.4	90, 15.1	24, 12.1
*TP53*	87, 11.0, 27.0^a^	44, 9.5	43, 13.0	64, 10.8	23, 11.6
*CCND1*	31, 3.9, 9.6^a^	21, 4.5	10, 3	22, 3.7	9, 4.5
*KMT2D*	31, 3.9, 9.6^a^	28, 6.1	3, 0.9	23, 3.9	8, 4.0
*NSD2*	26, 3.3, 8.1^a^	17, 3.7	9, 2.7	20, 3.4	6, 3.0
Number of somatic mutations, median, range, genes	5 (0–12)	2 (0–10)	2 (0–12)	2 (0–12)	2 (0–10)
Translocation (11:14), *n*, %	480, 60.5, 77.4^b^	290, 62.8	190, 57.2	356, 60.0	124, 6.2
Complex karyotype, *n*, %	78, 9.8, 12.6^b^	26, 5.6	52, 15.7	58, 9.7	20, 10
Deletion chromosome Y, *n*, % male	47, 5.9, 10.6^b^	24, 7.2	23, 9.1	37, 6.2	10, 5.0
Deletion chromosome 17, *n*, %	81, 10.2, 13.1^b^	39, 5.2	42, 6.9	55, 9.2	26, 13.0

^a^Percentage in patients with genomic data. (*n*/322).

^b^Percentage in patients with cytogenetic data. (*n*/620); males (*n*/445).

### Optimized Hyperparameters and XGBoost Metrics

**FIGURE 2:**
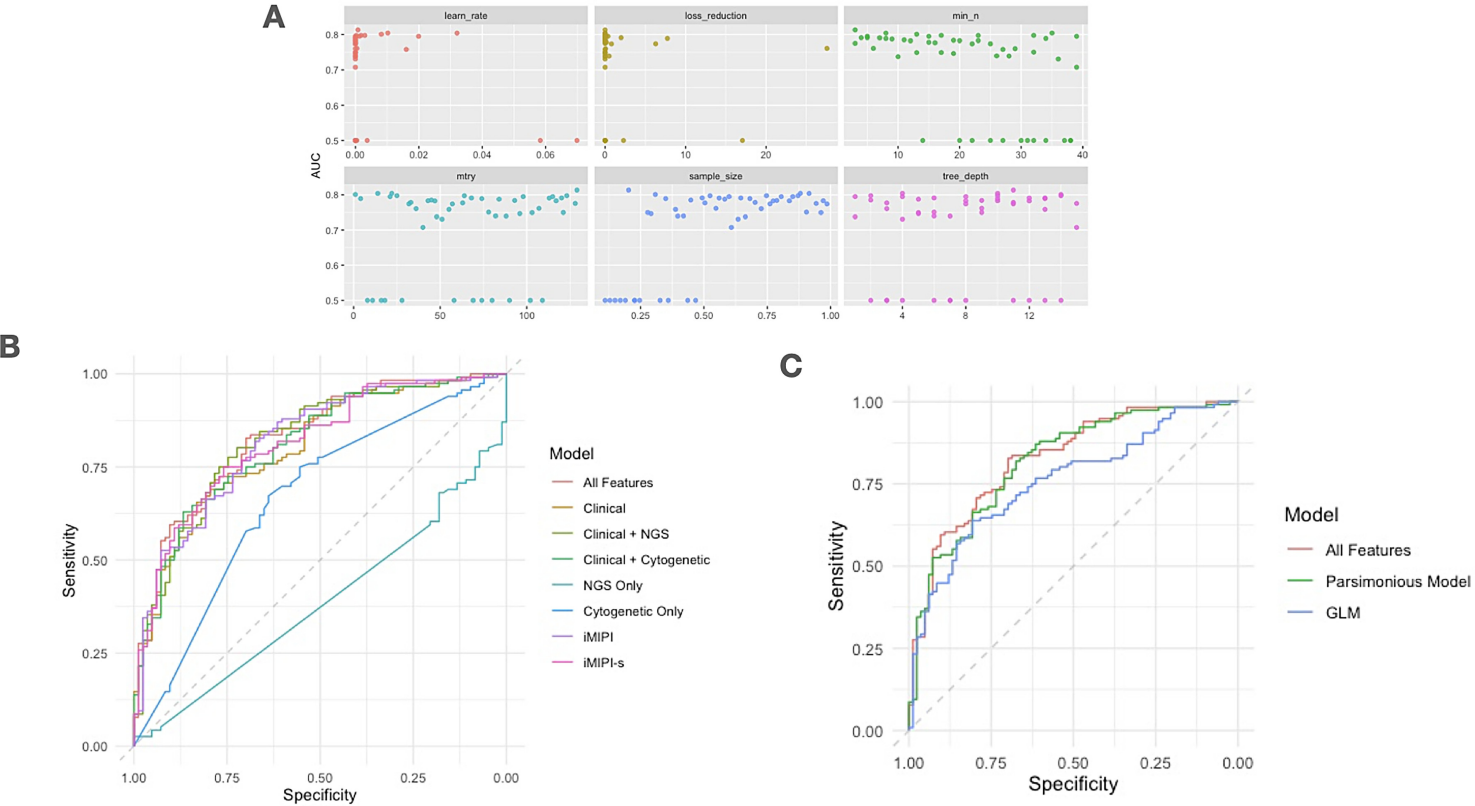
Hyperparameter fitting and model metrics. **A,** A scatterplot showing the corresponding mean AUC (from cross-fold validation sets) to hyperparameter values tuned by the 50-grid Latin hypercube on the “all features” XGBoost model. **B,** Comparison of ROC curves from XGBoost Models. Of the trained models, the “all features” model performed the best on the test set, followed by the two “parsimonious” models (the iMIPI and iMIPI-s) and the combined “clinical and NGS” and “clinical and cytogenetic models.” **C,** Comparison of ROC curves from the “all features” and “parsimonious” (20-feature) XGBoost models and the multivariate linear model (GLM).

A representation of the possible hyperparameter fits using the ROC AUC for the “all features” model is shown in Fig. 2A, and all models are shown in Supplementary Fig. S1. A summary of the hyperparameters that yielded the highest ROC AUC for the XGBoost models is presented in Supplementary Table S4.

Of the four XGBoost models fit, the “all features model” containing all of the feature types performed the best on our training and test sets (Table 2). The ROC AUC for the test set was 0.83, with an accuracy of 0.77. In addition, the model yielded a sensitivity of 68.7% and specificity of 81.9%. The Clinical + NGS model also performed well on the test set with a ROC AUC of 0.82 and an accuracy of 0.76. Sensitivity was higher with this model (72.3%), demonstrating its ability to predict aggressive cases with clinical and genomic information. However, this model yielded more false positives than the “all features” model (specificity = 78.5%).

**TABLE 2:** Performance metrics of prognostic MCL models

Model	Features	Preprocessed	AUC Train	AUC Test	Accuracy	Sensitivity	Specificity	PPV	NPV	PLR	F1
XGB all features	129	64	0.81	0.83	0.77	0.69	0.82	0.73	0.79	3.79	0.71
XGB parsimonious “iMIPI”^a^	20	24	0.82	0.82	0.75	0.70	0.77	0.68	0.78	3.00	0.69
XGB parsimonious “iMIPI-s”^b^	10	12	0.81	0.81	0.74	0.67	0.78	0.68	0.77	3.01	0.68
XGB complete cyto case	128	65	0.84	0.80	0.75	0.59	0.89	0.76	0.78	5.18	0.67
XGB clinical + cyto	86	99	0.79	0.81	0.73	0.67	0.74	0.65	0.76	2.61	0.66
XGB clinical + NGS	67	80	0.82	0.82	0.76	0.72	0.78	0.71	0.80	3.35	0.71
XGB clinical only	24	37	0.79	0.80	0.71	0.68	0.75	0.66	0.76	2.70	0.67
XGB cyto only	62	62	0.64	0.67	0.66	0.49	0.78	0.61	0.68	2.20	0.55
XGB NGS only	43	43	0.67	0.61	0.57	0.81	0.40	0.49	0.74	1.34	0.61
Random forest	20	21	0.78	0.79	0.72	0.64	0.78	0.68	0.76	2.70	0.67
GLM	20	24	0.74	0.77	0.72	0.61	0.79	0.68	0.74	2.97	0.65
MIPI^c^	1	1	0.62	0.64	0.60	0.30	0.81	0.53	0.62	1.59	0.39
MIPIb^d^	1	1	0.66	0.66	0.60	0.27	0.84	0.54	0.61	1.62	0.35
Cox PH (OS)	20	20	NA	0.76^e^	NA	NA	NA	NA	NA	NA	NA
Cox PH (OS)	10	10	NA	0.75^e^	NA	NA	NA	NA	NA	NA	NA
Cox PH (PFS)	20	20	NA	0.73^e^	NA	NA	NA	NA	NA	NA	NA
Cox PH (PFS)	10	10	NA	0.71^e^	NA	NA	NA	NA	NA	NA	NA

Abbreviations: AUC, area under the receiver operating characteristic curve; cyto, cytogenetic; F1, F1 score; GLM, generalized linear model—a multivariate logistic model; NGS, next-generation sequencing; NPV, negative predictive value; PLR, positive likelihood ratio; PPV, positive predictive value; XGB, XGBoost.

^a^iMIPI = proposed integrated mantle cell lymphoma international prognostic index. Model launched as REST API.

^b^iMIPI = proposed integrated mantle cell lymphoma international prognostic index, simplified. Model launched as REST API.

^c^MIPI = mantle cell lymphoma international prognostic index. Constructed from LDH/ULN, age at diagnosis, and ECOG performance status.

^d^MIPIb = Biological MIPI – adds Ki-67%, a proliferation index.

^e^A concordance index (C) is used instead of AUC for PH models.

The parsimonious models performed well. The 20-feature parsimonious model had a ROC AUC of 0.82 on the training set and 0.82 on the test set. Sensitivity was higher than the “all features” model (70%), and specificity was slightly lower (76.7%). The 10-feature parsimonious model had a slightly lower ROC AUC on the training and test sets (0.81) than the 20-feature model; however, the sensitivity of the most sparse model was lower (67%) with slightly higher specificity (78%).

The metrics and combined ROC curves of the XGBoost classification models are presented in Table 2 and Fig. 2B. Supplementary Table S2 lists the features of the models that were included and removed.

### Variable Importance and SHAP Additive Explanations

**FIGURE 3:**
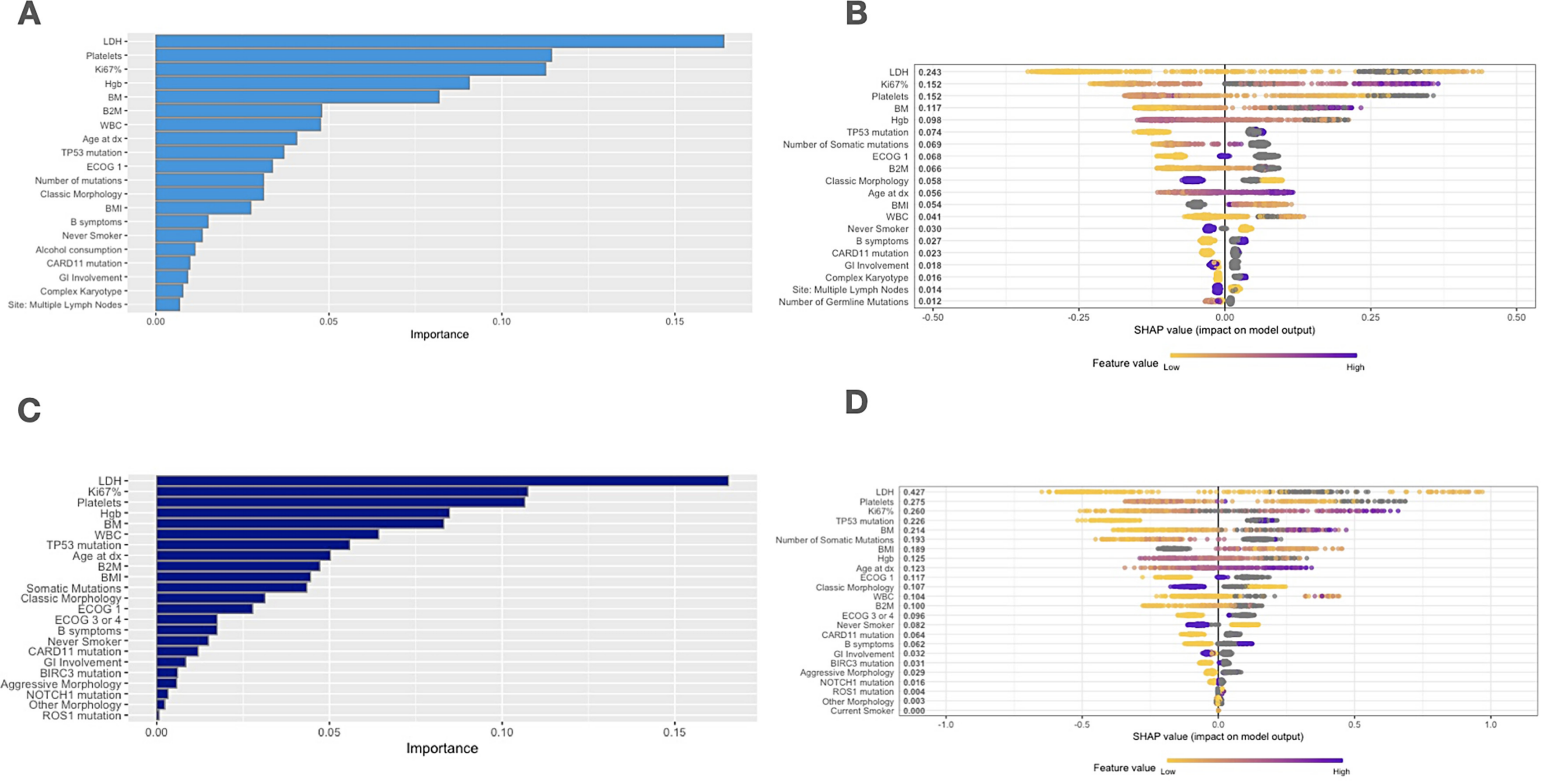
Feature importance in the combined feature XGBoost models. **A,** The VIP for the top 20 features from the “all features” XGBoost model. B2M, beta-2 microglobulin; BM, bone marrow involvement; BMI, body mass index; dx, diagnosis; ECOG, Eastern Cooperative Oncology Group performance status; GI, gastrointestinal; Hgb, hemoglobin; LDH, lactase dehydrogenase; WBC, white blood cell count. **B,** SHAP additive values demonstrating feature impact on model output from the “all features” XGBoost model. Colors represent the value of the feature; note continuous features have a color gradient, while the dummy variables values of 0 and 1 separate into two groups “low and high”. **C,** The VIP for features that were included in the parsimonious model or iMIPI. The top 20 features were selected from the most important features from the “all features” model; 23 features are represented in the figure due to categorical variables being transformed into dummy variables during data preprocessing. **D,** SHAP values from the parsimonious model (iMIPI).

VIPs and SHAP values from the full XGBoost model and the parsimonious model for the top features are shown in Fig. 3. The SHAP values from other XGBoost models are shown in Supplementary Fig. S3. LDH, Ki-67%, platelet count, bone marrow involvement, beta-2 microglobulin (B2M), WBC count, age at diagnosis, and *TP53* mutational status were important features in the models. Morphology and modifiable exposure factors such as smoking status and alcohol consumption (drinks per week) were also impactful.

### Assessment of Split-finding Algorithm for Missing Data—Comparing a “complete case” Model

The model with patients who had full cytogenetic data was evaluated for performance. One feature had to be removed because of low variance (deletion of chromosome 14) when included in the smaller test set which contained 155 patients. The model performed well on the training set with an ROC AUC of 0.84; however, the model performed worse on the test set than the all features and parsimonious models constructed with full patient sets that had missing data (ROC AUC of 0.80 and accuracy of 0.75). This model also had lower sensitivity (59%) and high sensitivity (89%) demonstrating a lower ability to detect true positives (aggressive disease) and a higher ability to detect true negatives (mild/indolent disease). The metrics for the complete-case model is displayed in Table 2. VIP and SHAPs were slightly different for this model and included some features not represented in the parsimonious model (Supplementary Fig. S3).

### Random Forest Model

The random forest model performed fairly well, but not as well as the parsimonious 20-feature XGBoost model with an ROC AUC = 0.78 on the training set and 0.79 on the test set. The sensitivity was also much lower at 53%. Metrics of this model are in Table 2.

### GLM

The metrics, variable coefficients, SEs, and *P* values for the GLM are shown in Supplementary Table S4. The GLM did not perform as well as most of the XGBoost Models on the training or testing set with ROC AUC = 0.77, accuracy = 0.72, sensitivity = 61%, and specificity = 79% on the test set (Table 2). Ki-67%, total number of somatic mutations, LDH, aggressive morphology, and age at diagnosis were significant predictors of poor outcomes, whereas nonsmoking status was significant for predicting mild/responsive outcomes. The ROC curves for the GLM model, all feature models, and the parsimonious model are shown in Fig. 2C. Regression coefficients for the GLM are displayed in Supplementary Table S5.

### Survival Models and Kaplan–Meier Curves for Identified Features

The regression coefficients and HRs for the PFS and OS Cox PH models are shown in Table 3. The two survival models had concordance indices (C) of 0.76 for the OS model and 0.74 for the PFS model. Although not directly comparable, the XGBoost models with the same features (parsimonious models) exhibited a better performance (Table 2).

**TABLE 3:** Cox regression models

	Progression-free survival			Overall survival
Characteristic	HR^a^	SE	HR^a^	SE
Morphology				
Blastoid	—	—	—	—
Classic	1.28	0.205	1.44	0.266
Pleomorphic	2.03**	0.243	2.09*	0.293
Other	1.39	0.254	1.23	0.352
Ki-67%	1.51***	0.077	1.63***	0.102
Bone marrow involvement	1.15	0.071	1.07	0.094
Lactate dehydrogenase	1.24***	0.046	1.05	0.043
Beta-2 microglobulin	1.01	0.064	1.12	0.072
White blood cell count	1.02	0.047	1.11	0.054
Hemoglobin	0.92	0.074	0.82*	0.097
Platelets	0.92	0.061	0.92	0.079
ECOG				
0	—	—	—	—
1	1.04	0.147	1.16	0.196
2	1.65	0.521	3.10*	0.490
3	9.54***	0.627	6.89***	0.570
B-symptoms	1.42*	0.140	1.15	0.189
Smoking status				
Former	—	—	—	—
No	0.69**	0.129	0.62**	0.168
Yes	1.04	0.245	0.78	0.323
Age at diagnosis	1.18*	0.066	1.22*	0.089
BMI	0.94	0.067	0.91	0.091
*TP53* mutation	1.56**	0.140	1.26	0.193
*CARD11* mutation	0.53	0.434	0.76	0.540
Number of somatic mutations	1.11	0.068	1.12	0.094
*BIRC3* mutation	0.98	0.312	1.39	0.369
GI Involvement	1.32	0.160	1.45	0.233
*NOTCH1* mutation	0.77	0.519	0.85	0.733
*ROS1* mutation	1.04	0.358	0.57	0.631

Abbreviations: HR, hazard ratio; SE, standard error.

^a^*, *P* < 0.05; **, *P* < 0.01; ***, *P* < 0.001.

**FIGURE 4:**
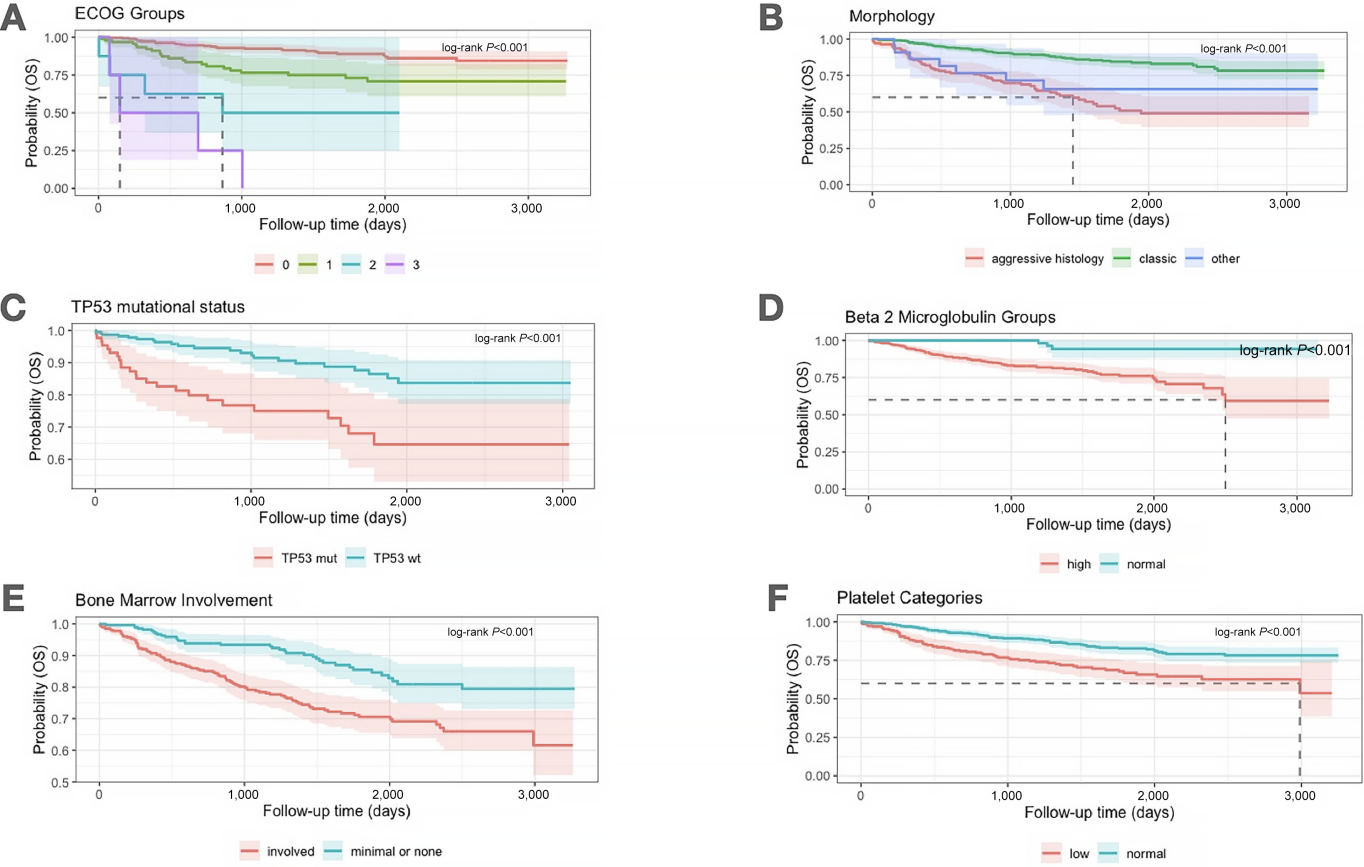
Selected Kaplan–Meier plots from top XGBoost model features. The median OS time is represented by the dashed line. Differences between groups determined by the log-rank test were statistically significant. **A,** OS in ECOG performance category groups assigned at baseline. **B,** OS in morphology groups identified from baseline biopsies. The aggressive histology group comprises blastoid, pleomorphic, and combined blastoid and pleomorphic morphologies. The “other” category represents classic histology biopsies with blastoid or pleomorphic features or unclassifiable biopsies. **C,** OS in *TP53* mutational status groups. The mutation was identified from baseline biopsy NGS. **D,** OS in B2M level category groups. The high B2M category was defined from B2M levels higher than 1.8 mcg/mL. **E,** OS in bone marrow involvement groups. “Involved” bone marrow was considered to be higher than 5% MCL bone marrow involvement in a baseline sample. **F,** OS in platelet count groups. Low platelets were defined as lower than 150,000 per μL.

Selected Kaplan–Meier curves for OS and PFS from the top XGBoost model identified features are shown in Fig. 4. The XGBoost model identified features that had significantly different OS and PFS in the two classified groups (Supplementary Table S6). Notable findings were significant differences in OS and PFS were Ki-67%, morphology groups (aggressive histology vs. classic), TP53 mutational status, B2M, bone marrow involvement, platelet count, smoking status, and BMI. Total mutation count (greater than 5) was not found to significantly impact survival.

### Comparison with Existing Prognostic Indices (MIPI)

Metrics for the univariate MIPI models were substantially lower than those of the multivariate ML, GLM, and Cox models (MIPI: ROC AUC = 0.64, accuracy = 0.6; MIPI-b: ROC AUC = 0.66, accuracy = 0.6; Table 2). Our combined XGBoost model with 129 features had an AUC improvement on the test set of 0.19 for the MIPI and 0.17 for the MIPI-b. The parsimonious XGBoost model with 20 features had similarly improved AUC (0.18 for MIPI and 0.16 for MIPI-b). The parsimonious model also had improved accuracy to the existing MIPIs (0.75 vs. 0.6).

### The Integrative MIPI

We designated our 20-feature ML parsimonious model as the integrative MIPI or (i-MIPI). The index combines the following features: LDH, platelet count, Ki-67% hgb, bone marrow involvement, B2M, WBC, age at diagnosis, *TP53, CARD11, BIRC3, NOTCH1 and ROS1* mutational status, ECOG status, number of somatic mutations, morphology, BMI, B-symptoms, smoking status, gastrointestinal involvement. The performance metrics of the i-MIPI are the same as our parsimonious model on both our training and tests sets and yielded an AUC of 0.82 for both datasets. Because *ROS1* and *NOTCH1* mutational status are lowest in importance, it would be prudent to assess their impact on prognoses as more NGS studies are conducted.

In addition, we named our 10-feature parsimonious model the i-MIPI simplified or i-MIPI-s. Although lower in sensitivity, clinical utility is improved as the model is based on only 10 features: LDH, platelet count, Ki-67% hgb, bone marrow involvement, B2M, *TP53* mutational status, ECOG status (1 or >2), number of somatic mutations, and morphology (classic, blastoid, or pleomorphic).

### REST API—Clinical Implementation

Fitting a full XGBoost model requires multiple steps, and a moderate amount of computational time and power. To address these factors, the parsimonious XGBoost models: i-MIPI and i-MIPIs were versioned and deployed as proof of concept using a REST API, demonstrating clinical utility.

Clinicians or researchers can enter some or all of the 10 or 20 model features for a patient as the XGBoost model can make predictions with missing data. The REST API predicts disease classes (aggressive vs. responsive/indolent) using the launched ML model, provides the probability of an accurate prediction based on available data, and monitors the model. Future studies should inform and dynamically update the model especially as more research is done regarding the prognostic impact of mutations.

## Discussion

This is the largest patient cohort to be used in an integrative prognostic model of MCL. The XGBoost ML classification models used in our large cohort exhibited superior performance compared with traditional statistical models. Suitably, combining the robust feature selection aspect of XGBoost with GLM and Cox survival models allowed the generation of statistics such as *P* values from regression coefficients and odds, HRs, and KM curves that could be easily interpreted. Survival analyses were tailored using the results from the ML models and confirmed prior literature regarding impactful baseline characteristics on patient outcomes.

Among the combined feature-type models [all features, parsimonious (10 and 20 feature), GLM, and Cox], LDH levels, Ki-67 proliferation index (%), blood counts (WBC, platelets, hemoglobin), B2M levels, bone marrow infiltration, and age at diagnosis were the most significant prognostic factors for MCL. However, other features encompassing genomic, cytogenetic, and clinicopathologic data were deemed important by the XGBoost models, including *TP53* mutational status and the total number of somatic mutations. This indicates that small contributions from multiple molecular and pathophysiologic sources contribute to therapeutic responses and outcomes. It is interesting that the most frequently mutated gene in MCL, *ATM,* was not represented as a top prognostic factor in any models. In addition, the composite feature generated from the total somatic mutation count may be acting as a surrogate measure of tumor mutational burden. Future studies may integrate this measure as a more precise representation of mutational frequency. Features derived from NGS were superior to cytogenetic features in contributing to prognostic impact demonstrating the value of more granular molecular studies. As NGS is further implemented into clinical practice, cytogenetic studies may become less important for diagnosis and prognosis.

Clinical biomarkers such as Ki-67%, a cellular proliferation marker, and B2M represent more aggressive and advanced disease at baseline and are important in predicting prognoses when combined with molecular features. Platelet and WBC count typically represent the hallmarks of lymphoma on bone marrow function and remain important prognostic indicators.

In addition, the XGBoost models were able to represent nonlinear relationships among multiple features and outcomes more accurately than traditional linear models. The gradient-boosted framework also performed better than a random forest models with identical data. Our model uniquely identified impactful and potentially modifiable features of the clinical exposome: BMI, smoking status, and alcohol consumption. Further research will be conducted to understand the significance of these features in combination with genomic and transcriptomic interactions and MCL prognoses.

Our cohort had incomplete genomic and cytogenetic data, especially for patients diagnosed with MCL in earlier years. We assessed the ability of the XGBoost sparsity aware split-finding algorithm utilizing a complete-cytogenetic case model where we included only patients with complete cytogenetic data. The model performed comparably well on the training set, but had a decrease in performance when fit on the test set indicating possible overfitting and lower generalizability. The model did signify other important features when assessing VIP and SHAP, but only included one cytogenetic feature (complex karyotype) indicating possible representation of cytogenetic processes in other biological features. Building a model with missing data did not reduce model performance and is perhaps more generalizable in other clinical settings where data for some features have not been collected. We hypothesize that model performance would be better with more complete data for all 862 patients, especially from NGS. Mutations included in the XGBoost models may change and be updated with further investigations.

We were also limited by the lack of an external validation cohort for our models. We had access to the full EMR which enhanced our ability to extract several baseline clinicopathologic features; publicly available MCL datasets lack the granular feature information needed to make a suitable comparison. To ameliorate this limitation, our large dataset was split randomly to ensure a robust training and test set. In addition, the training set was divided into 10 folds for cross-validation resampling to further enhance generalizability. Future validation of our model will include external validation from new data or outside institutional cohorts.

ML models allow for the inclusion and interpretation of many more variables than models that use traditional statistical methods and should be incorporated into correlative genomic studies of MCL and other rare malignancies. Although our cohort was remarkably large for rare diseases, feature selection using ML may be valuable in multi-omic studies of cancers with smaller sample sizes and moderate amounts of missing data.

The actual predictive power of our XGBoost model may be slightly attenuated as patients recently treated may yet relapse. We considered it important to include all patients in our dataset with a year-of-diagnosis criterion to avoid overrepresentation bias of aggressive cases. To explore the censored time aspect of MCL, the Cox models considered various lengths of follow-up time. XGBoost with censored regression has been developed but is used infrequently as performance metrics, concordance indices, and variable importance elements are challenging to interpret.

In future studies, we will incorporate other -omics data, especially from RNA sequencing (RNA-seq), to provide insight into the functional impact of DNA mutations. We look forward to integrating pathway analysis in RNA-seq, whole-genome sequencing, and whole-exome sequencing as feature extraction tools for future models. Another opportunity is to implement temporal measures in time-series ML models with circulating tumor DNA to evaluate the effect of minimal residual disease and clonal evolution on response to therapy. We also foresee the potential of incorporating these techniques to stratify patients in precision medicine trials.

ML techniques similar to those demonstrated in this study allow for thousands of features to be incorporated into algorithms and return variable importance for the most impactful biomarkers. The proposed i-MIPI is intended to be dynamic and updated via ML as new molecular and clinical studies in MCL emerge. Currently, our model can only provide a binary prediction. However, in the future, we aim to enhance its capability to generate more precise prognostic predictions. As additional correlative clinical multi-omic studies become available, matched therapeutics may be incorporated into the algorithm. Although past indices were useful in stratifying patients at baseline with limited biomarkers, the integration of multiple features derived from multi-omics and clinical measurements will enhance future prognostic applications.

## Supplementary Material

Supplementary Data Description Supplementary Data Description

Supplementary Table 1 Initial Treatment List for Mantle Cell Lymphoma Patients

Supplementary Table 2 Full feature list with abbreviations from the dataset

Supplementary Table 3 Missingness among Full Dataset Features

Supplementary Table 4 Hyperparameters for XGBoost Models

Supplementary Table 5 Coefficients from the Multivariate Generalized Linear Model

Supplementary Table 6 Univariate survival comparisons from features identified from XGBoost.

Supplementary Figure 1 S1. Hyperparameter tuning of other XGBoost models

Supplementary Figure 2 S2. Variable Importance from Full XGBoost Model (training cross-validation sets)

Supplementary Figure 3 S3. Feature Importance from other XGBoost models

Supplementary Figure 4 S4. Correlation Plot of Numeric Features

Supplementary Methods Supplementary detailed methods
