# Supplementary material for: Integrative Prognostic Machine Learning Models in Mantle Cell Lymphoma
Source: Cancer Res Commun. 2023 Aug 2;3(8):1435–46. doi: 10.1158/2767-9764.CRC-23-0083 (PMC10395375; doi:10.1158/2767-9764.CRC-23-0083)
Supplement: Supplementary Data Description [file crc-23-0083-s01.pdf]

Supplementary Materials

Supplementary Table 1: Initial Treatment List for Mantle Cell Lymphoma Patients (n=794).

Supplementary Table 2: Full feature list with abbreviations from the dataset
Features (variables) shaded in gray were removed either for low variance or representation in the respective outcome classes.

A. Features from clinicopathologic data
B. Features from cytogenetic data
C. Features from NGS data (targeted panels and WES)
D. Other features included in statistical models (survival and univariate MIPIs)

Supplementary Table 3: Missingness among Full Dataset Features
Missingness among patient features included in the "all feature type" model.

Supplementary Table 4: Hyperparameters for XGBoost Models
Optimal hyperparameters as determined by the highest ROC AUC for each type of model.

Supplementary Table 5: Coefficients from the Multivariate Generalized Linear Model

Supplementary Table 6: Univariate survival comparisons from features identified from XGBoost. The log-rank test was used to compare overall survival (OS) and progression-free survival (PFS) between groups. – p.26

Supplementary Figure 1: Hyperparameter tuning of other XGBoost models
A: Hyperparameter fit of clinical data only XGBoost model
B: Hyperparameter fit of clinical + cytogenetic data XGBoost model
C: Hyperparameter fit of clinical + NGS data XGBoost model
D: Hyperparameter fit of cytogenetic data only XGBoost model
E: Hyperparameter fit of NGS data only XGBoost model

Supplementary Figure 2: Variable Importance from Full XGBoost Model (training cross-validation sets) Variable importance for feature selection was determined from the 10 cross-fold validation sets that came from the training data. The optimized hyperparameters were fit on the folds and the variable importance was determined.

Supplementary Figure 3: Feature Importance from other XGBoost models

A. The VIP for the top 30 features that were included in the NGS-only model.
B. SHAP values for the top 20 features included in the NGS-only model.
C. The VIP for the top 20 features that were included in the cytogenetic-only model.
D. SHAP values for the top 20 features included in the cytogenetic-only model.
E. The VIP for the top 20 features that were included in the clinical + cytogenetic model.
F. SHAP values for the top 20 features included in the clinical + cytogenetic model.
G. The VIP for the top 20 features that were included in the clinical + NGS model.

H. SHAP values for the top 20 features included in the clinical + NGS model

I. SHAP values for model using more complete data. All patients had full cytogenetic data.

J. The VIP for model using more complete data. All patients had full cytogenetic data.

Supplementary Figure 4: Correlation Plot of Numeric Features.
An examination of complete pairwise Pearson's Product-Moment Correlation (r) between numeric variables in the full dataset.

Extended Methods: In-depth description of data curation, cytogenetic and sequencing methods.
