# Supplementary material for: Integrative Prognostic Machine Learning Models in Mantle Cell Lymphoma
Source: Cancer Res Commun. 2023 Aug 2;3(8):1435–46. doi: 10.1158/2767-9764.CRC-23-0083 (PMC10395375; doi:10.1158/2767-9764.CRC-23-0083)
Supplement: Supplementary Table 1 — Initial Treatment List for Mantle Cell Lymphoma Patients [file crc-23-0083-s02.pdf]

Supplementary Table 1: First Therapy in MCL patients (n=794)

First Treatment	Aggressive (n=332)	Indolent / Responsive (n=462)
Auto-SCT	19	13
Allo-SCT	5	1
r-CHOP	32	7
r-CHOP + other therapy	8	2
r-CHOP x r-DHAP	13	5
r-HCVAD	44	22
*r-Bendamustine**	81	78
*r-Ibrutinib***	58	178
r-Acalabrutinib	2	29
r-Ibrutinib + venetoclax	9	48
r-Acalabrutinib + venetoclax	5	4
BTKi (other)	3	12
*Rituximab / lenalidomide***	29	19
Radiation	3	0
Other Chemotherapies	11	8
Untreated / Observation / Missing	10	36

Auto = autologous; Allo = allogenic; SCT = stem cell transplant
r = rituximab
CHOP =cyclophosphamide, doxorubicin, vincristine
DHAP = dexamethasone, cytarabine, cisplatin
HCVAD = hyper-fractionated cyclophosphamide, vincristine, doxorubicin hydrocholoride
BTKi = Bruton's tyrosine kinase inhibitor

**Includes added BTKi in some clinical studies*
*** Some regimens include radiation*
