# Supplementary material for: Integrative Prognostic Machine Learning Models in Mantle Cell Lymphoma
Source: Cancer Res Commun. 2023 Aug 2;3(8):1435–46. doi: 10.1158/2767-9764.CRC-23-0083 (PMC10395375; doi:10.1158/2767-9764.CRC-23-0083)
Supplement: Supplementary Table 2 — Full feature list with abbreviations from the dataset [file crc-23-0083-s03.pdf]

Supplementary Table 2 - Full Feature List

Feature	Abbreviation	Source	Type	Description	Measurement	Values (range)	removed NZV
Sex		clinical	binary	biological sex	medical history	male, female	
Race		clinical	categorical	race and or ethnicity	self-report	White, Black, Asian, Hispanic, Native American or Alaskan native, other/unknown	
Morphology	morph	pathology	categorical	tumor morphology	pathology study	classic, blastoid, pleomorphic, leukemic, other aggressive histology	
Pattern		pathology	categorical	histological pattern	pathology study	interstitial, nodular, diffuse, unusual_other (combinations)	
Ki 67 (%)	ki_67	pathology	continuous	proliferation marker percentage			
Bone marrow involvment (%)	bm	pathology	continuous	percentage of bone marrow involved	pathology study		
GI Involment	gi_involve	pathology	binary	MCL present in gastrointestinal biopsy			
Lactase Dehydrogenase	ldh	clinical	continuous				
Beta2 Microglobulin	b2m	clinical	continuous				
White blood cell / leukocyte	wbc	clinical	continuous				
Hemoglobin	hgb	clinical	continuous				
Platelets	platelets	clinical	continuous				
ECOG status	ecog	clinical	categorical				
B-symptoms	b_symptom	clinical	binary	B-symptoms: weight loss, nightsweats, fever	self-report	yes, no, missing	
Stage		clinical	categorical				YES
Site		clinical	categorical				
Body mass index (BMI)	bmi	clinical_exposure	continuous		medical history		
Smoking status	smoke	clinical_exposure	categorical		self-report		
Smokless tobacco use	smokeless	clinical_exposure	categorical		self-report		
Alcohol use	alcohol	clinical_exposure	continuous	drinks per week	self-report		
Prior cancer diagnosis	prior_cancer	clinical_exposure	binary		medical history		
Prior chemotherapy	prior_chemo	clinical_exposure	binary		medical history		
Prior radiation	prior_radiation	clinical_exposure	binary		medical history		
Bulky disease	bulky	clinical	binary		radiology report		
Age at diagnosis	age_dx_years	clinical	continuous	Interval between date of birth and date of diagnosis	medical history		

Feature	Chromosome	Abbreviation	Source	Type
Translocation	11:14	t_11:14	cytogenetic	binary
Translocation	14:18	t_14:18	cytogenetic	binary
Deletion	1	del_1	cytogenetic	binary
Deletion	2	del_2	cytogenetic	binary
Deletion	3	del_3	cytogenetic	binary
Deletion	4	del_4	cytogenetic	binary
Deletion	5	del_5	cytogenetic	binary
Deletion	6	del_6	cytogenetic	binary
Deletion	7	del_7	cytogenetic	binary
Deletion	8	del_8	cytogenetic	binary
Deletion	9	del_9	cytogenetic	binary
Deletion	10	del_10	cytogenetic	binary
Deletion	11	del_11	cytogenetic	binary
Deletion	12	del_12	cytogenetic	binary
Deletion	13	del_13	cytogenetic	binary
Deletion	14	del_14	cytogenetic	binary
Deletion	15	del_15	cytogenetic	binary
Deletion	16	del_16	cytogenetic	binary
Deletion	17	del_17	cytogenetic	binary
Deletion	18	del_18	cytogenetic	binary
Deletion	19	del_19	cytogenetic	binary
Deletion	20	del_20	cytogenetic	binary
Deletion	21	del_21	cytogenetic	binary
Deletion	22	del_22	cytogenetic	binary
Deletion	X	del_x	cytogenetic	binary
Deletion	Y	del_y	cytogenetic	binary
Addition	1	add_1	cytogenetic	binary
Addition	2	add_2	cytogenetic	binary
Addition	3	add_3	cytogenetic	binary
Addition	4	add_4	cytogenetic	binary
Addition	5	add_5	cytogenetic	binary
Addition	6	add_6	cytogenetic	binary
Addition	7	add_7	cytogenetic	binary
Addition	8	add_8	cytogenetic	binary
Addition	9	add_9	cytogenetic	binary
Addition	10	add_10	cytogenetic	binary
Addition	11	add_11	cytogenetic	binary
Addition	12	add_12	cytogenetic	binary
Addition	13	add_13	cytogenetic	binary
Addition	14	add_14	cytogenetic	binary
Addition	15	add_15	cytogenetic	binary
Addition	16	add_16	cytogenetic	binary
Addition	17	add_17	cytogenetic	binary
Addition	18	add_18	cytogenetic	binary
Addition	19	add_19	cytogenetic	binary
Addition	20	add_20	cytogenetic	binary

Addition	21	add_21	cytogenetic	binary
Addition	22	add_22	cytogenetic	binary
Addition	ring	add_r	cytogenetic	binary
Addition	sex	add_sex	cytogenetic	binary
Other	1	other_1	cytogenetic	binary
Other	2	other_2	cytogenetic	binary
Other	3	other_3	cytogenetic	binary
Other	4	other_4	cytogenetic	binary
Other	5	other_5	cytogenetic	binary
Other	6	other_6	cytogenetic	binary
Other	7	other_7	cytogenetic	binary
Other	8	other_8	cytogenetic	binary
Other	9	other_9	cytogenetic	binary
Other	10	other_10	cytogenetic	binary
Other	11	other_11	cytogenetic	binary
Other	12	other_12	cytogenetic	binary
Other	13	other_13	cytogenetic	binary
Other	14	other_14	cytogenetic	binary
Other	15	other_15	cytogenetic	binary
Other	16	other_16	cytogenetic	binary
Other	17	other_17	cytogenetic	binary
Other	18	other_18	cytogenetic	binary
Other	19	other_19	cytogenetic	binary
Other	20	other_20	cytogenetic	binary
Other	21	other_21	cytogenetic	binary
Other	22	other_22	cytogenetic	binary
Other	sex	other_sex	cytogenetic	binary
Dual TP53 loss	17	tp53_loss	cytogenetic	binary
Tetraploid Population	all	tetraploid	cytogenetic	binary
Complex Karyotype	all	complex_kar	cytogenetic	binary
Abnormal Karyotype	all	abrnormal_kar	cytogenetic	binary

Measurement	Values (range)	removed (NZV)	removed (class imbalance)
FISH	yes, no, missing		
FISH	yes, no, missing		
FISH	yes, no, missing		
FISH	yes, no, missing		
FISH	yes, no, missing		
FISH	yes, no, missing		
FISH	yes, no, missing		YES
FISH	yes, no, missing		
FISH	yes, no, missing		
FISH	yes, no, missing		
FISH	yes, no, missing		
FISH	yes, no, missing		
FISH	yes, no, missing		
FISH	yes, no, missing		
FISH	yes, no, missing		
FISH	yes, no, missing		
FISH	yes, no, missing		
FISH	yes, no, missing		
FISH	yes, no, missing		
FISH	yes, no, missing		
FISH	yes, no, missing		
FISH	yes, no, missing		
FISH	yes, no, missing		
FISH	yes, no, missing		
FISH	yes, no, missing		
FISH	yes, no, missing		
FISH	yes, no, missing		
FISH	yes, no, missing		
FISH	yes, no, missing		
FISH	yes, no, missing		
FISH	yes, no, missing		
FISH	yes, no, missing		
FISH	yes, no, missing		
FISH	yes, no, missing		
FISH	yes, no, missing		
FISH	yes, no, missing		
FISH	yes, no, missing		
FISH	yes, no, missing		
FISH	yes, no, missing		
FISH	yes, no, missing		
FISH	yes, no, missing		
FISH	yes, no, missing		
FISH	yes, no, missing		
FISH	yes, no, missing		
FISH	yes, no, missing		
FISH	yes, no, missing		YES

FISH	yes, no, missing		
FISH	yes, no, missing		
FISH	yes, no, missing		YES
FISH	yes, no, missing	YES	
FISH	yes, no, missing		
FISH	yes, no, missing		
FISH	yes, no, missing		
FISH	yes, no, missing		
FISH	yes, no, missing		
FISH	yes, no, missing		
FISH	yes, no, missing		
FISH	yes, no, missing		
FISH	yes, no, missing		
FISH	yes, no, missing		YES
FISH	yes, no, missing		
FISH	yes, no, missing		YES
FISH	yes, no, missing		
FISH	yes, no, missing		
FISH	yes, no, missing		
FISH	yes, no, missing		
FISH	yes, no, missing		
FISH	yes, no, missing	YES	
FISH	yes, no, missing	YES	
FISH	yes, no, missing	YES	
FISH	yes, no, missing	YES	
FISH	yes, no, missing		
FISH	yes, no, missing		
FISH	yes, no, missing		YES
FISH	yes, no, missing		
FISH - engineered	yes, no, missing		
FISH - engineered	yes, no, missing		

Feature	Gene	Source	Type	Description	Measurement	Values (range)	removed (NZV)	removed (class imbalance)
somatic mutation	*ANKRD26*	genomic	binary	any identified, filtered somatic mutation in the gene	NGS panel or WES	yes, no, missing	YES	
somatic mutation	*ARID1A*	genomic	binary	any identified, filtered somatic mutation in the gene	NGS panel or WES	yes, no, missing		
somatic mutation	*ARID1B*	genomic	binary	any identified, filtered somatic mutation in the gene	NGS panel or WES	yes, no, missing		YES
somatic mutation	*ASXL1*	genomic	binary	any identified, filtered somatic mutation in the gene	NGS panel or WES	yes, no, missing		
somatic mutation	*ASXL2*	genomic	binary	any identified, filtered somatic mutation in the gene	NGS panel or WES	yes, no, missing	YES	
somatic mutation	*ATM*	genomic	binary	any identified, filtered somatic mutation in the gene	NGS panel or WES	yes, no, missing		
somatic mutation	*B2M*	genomic	binary	any identified, filtered somatic mutation in the gene	NGS panel or WES	yes, no, missing	YES	
somatic mutation	*BANK1*	genomic	binary	any identified, filtered somatic mutation in the gene	NGS panel or WES	yes, no, missing		YES
somatic mutation	*BAZ2A*	genomic	binary	any identified, filtered somatic mutation in the gene	NGS panel or WES	yes, no, missing	YES	
somatic mutation	*BCL10*	genomic	binary	any identified, filtered somatic mutation in the gene	NGS panel or WES	yes, no, missing	YES	
somatic mutation	*BCL2*	genomic	binary	any identified, filtered somatic mutation in the gene	NGS panel or WES	yes, no, missing		YES
somatic mutation	*BCL6*	genomic	binary	any identified, filtered somatic mutation in the gene	NGS panel or WES	yes, no, missing	YES	
somatic mutation	*BCL7A*	genomic	binary	any identified, filtered somatic mutation in the gene	NGS panel or WES	yes, no, missing	YES	
somatic mutation	*BCOR*	genomic	binary	any identified, filtered somatic mutation in the gene	NGS panel or WES	yes, no, missing		
somatic mutation	*BCOR1*	genomic	binary	any identified, filtered somatic mutation in the gene	NGS panel or WES	yes, no, missing	YES	
somatic mutation	*BIRC3*	genomic	binary	any identified, filtered somatic mutation in the gene	NGS panel or WES	yes, no, missing		
somatic mutation	*BLNK*	genomic	binary	any identified, filtered somatic mutation in the gene	NGS panel or WES	yes, no, missing	YES	
somatic mutation	*BRAF*	genomic	binary	any identified, filtered somatic mutation in the gene	NGS panel or WES	yes, no, missing	YES	
somatic mutation	*BRCC3*	genomic	binary	any identified, filtered somatic mutation in the gene	NGS panel or WES	yes, no, missing	YES	
somatic mutation	*BRD4*	genomic	binary	any identified, filtered somatic mutation in the gene	NGS panel or WES	yes, no, missing		
somatic mutation	*BRNP3*	genomic	binary	any identified, filtered somatic mutation in the gene	NGS panel or WES	yes, no, missing	YES	
somatic mutation	*BTG1*	genomic	binary	any identified, filtered somatic mutation in the gene	NGS panel or WES	yes, no, missing	YES	
somatic mutation	*BTK*	genomic	binary	any identified, filtered somatic mutation in the gene	NGS panel or WES	yes, no, missing		
somatic mutation	*CALR*	genomic	binary	any identified, filtered somatic mutation in the gene	NGS panel or WES	yes, no, missing	YES	
somatic mutation	*CARD11*	genomic	binary	any identified, filtered somatic mutation in the gene	NGS panel or WES	yes, no, missing		
somatic mutation	*CBL*	genomic	binary	any identified, filtered somatic mutation in the gene	NGS panel or WES	yes, no, missing	YES	
somatic mutation	*CBLB*	genomic	binary	any identified, filtered somatic mutation in the gene	NGS panel or WES	yes, no, missing	YES	
somatic mutation	*CBLC*	genomic	binary	any identified, filtered somatic mutation in the gene	NGS panel or WES	yes, no, missing	YES	
somatic mutation	*CCND1*	genomic	binary	any identified, filtered somatic mutation in the gene	NGS panel or WES	yes, no, missing		
somatic mutation	*CCND3*	genomic	binary	any identified, filtered somatic mutation in the gene	NGS panel or WES	yes, no, missing	YES	
somatic mutation	*CCR4*	genomic	binary	any identified, filtered somatic mutation in the gene	NGS panel or WES	yes, no, missing	YES	
somatic mutation	*CCR7*	genomic	binary	any identified, filtered somatic mutation in the gene	NGS panel or WES	yes, no, missing	YES	
somatic mutation	*CD274*	genomic	binary	any identified, filtered somatic mutation in the gene	NGS panel or WES	yes, no, missing	YES	
somatic mutation	*CD28*	genomic	binary	any identified, filtered somatic mutation in the gene	NGS panel or WES	yes, no, missing		YES
somatic mutation	*CD58*	genomic	binary	any identified, filtered somatic mutation in the gene	NGS panel or WES	yes, no, missing	YES	
somatic mutation	*CD79A*	genomic	binary	any identified, filtered somatic mutation in the gene	NGS panel or WES	yes, no, missing		YES
somatic mutation	*CD79B*	genomic	binary	any identified, filtered somatic mutation in the gene	NGS panel or WES	yes, no, missing		
somatic mutation	*CDKN2A*	genomic	binary	any identified, filtered somatic mutation in the gene	NGS panel or WES	yes, no, missing		YES
somatic mutation	*CDKN2B*	genomic	binary	any identified, filtered somatic mutation in the gene	NGS panel or WES	yes, no, missing	YES	
somatic mutation	*CEBPA*	genomic	binary	any identified, filtered somatic mutation in the gene	NGS panel or WES	yes, no, missing	YES	
somatic mutation	*CELSR3*	genomic	binary	any identified, filtered somatic mutation in the gene	NGS panel or WES	yes, no, missing		

somatic mutation	*CHD2*	genomic	binary	any identified, filtered somatic mutation in the gene	NGS panel or WES	yes, no, missing		YES
somatic mutation	*CIITA*	genomic	binary	any identified, filtered somatic mutation in the gene	NGS panel or WES	yes, no, missing		YES
somatic mutation	*CNOT3*	genomic	binary	any identified, filtered somatic mutation in the gene	NGS panel or WES	yes, no, missing	YES	
somatic mutation	*CREBBP*	genomic	binary	any identified, filtered somatic mutation in the gene	NGS panel or WES	yes, no, missing	YES	
somatic mutation	*CRLF2*	genomic	binary	any identified, filtered somatic mutation in the gene	NGS panel or WES	yes, no, missing	YES	
somatic mutation	*CSF3R*	genomic	binary	any identified, filtered somatic mutation in the gene	NGS panel or WES	yes, no, missing	YES	
somatic mutation	*CSMD3*	genomic	binary	any identified, filtered somatic mutation in the gene	NGS panel or WES	yes, no, missing		YES
somatic mutation	*CUX1*	genomic	binary	any identified, filtered somatic mutation in the gene	NGS panel or WES	yes, no, missing	YES	
somatic mutation	*CXCR4*	genomic	binary	any identified, filtered somatic mutation in the gene	NGS panel or WES	yes, no, missing		
somatic mutation	*DD3X*	genomic	binary	any identified, filtered somatic mutation in the gene	NGS panel or WES	yes, no, missing	YES	
somatic mutation	*DDX41*	genomic	binary	any identified, filtered somatic mutation in the gene	NGS panel or WES	yes, no, missing	YES	
somatic mutation	*DIS3*	genomic	binary	any identified, filtered somatic mutation in the gene	NGS panel or WES	yes, no, missing		YES
somatic mutation	*DNMT3A*	genomic	binary	any identified, filtered somatic mutation in the gene	NGS panel or WES	yes, no, missing		
somatic mutation	*DUSP2*	genomic	binary	any identified, filtered somatic mutation in the gene	NGS panel or WES	yes, no, missing	YES	
somatic mutation	*EED*	genomic	binary	any identified, filtered somatic mutation in the gene	NGS panel or WES	yes, no, missing	YES	
somatic mutation	*EGFR*	genomic	binary	any identified, filtered somatic mutation in the gene	NGS panel or WES	yes, no, missing		
somatic mutation	*EGR1*	genomic	binary	any identified, filtered somatic mutation in the gene	NGS panel or WES	yes, no, missing	YES	
somatic mutation	*EGR2*	genomic	binary	any identified, filtered somatic mutation in the gene	NGS panel or WES	yes, no, missing	YES	
somatic mutation	*ELANE*	genomic	binary	any identified, filtered somatic mutation in the gene	NGS panel or WES	yes, no, missing	YES	
somatic mutation	*ELF4*	genomic	binary	any identified, filtered somatic mutation in the gene	NGS panel or WES	yes, no, missing	YES	
somatic mutation	*EP300*	genomic	binary	any identified, filtered somatic mutation in the gene	NGS panel or WES	yes, no, missing		
somatic mutation	*ETNK1*	genomic	binary	any identified, filtered somatic mutation in the gene	NGS panel or WES	yes, no, missing	YES	
somatic mutation	*ETV6*	genomic	binary	any identified, filtered somatic mutation in the gene	NGS panel or WES	yes, no, missing	YES	
somatic mutation	*EWSR1*	genomic	binary	any identified, filtered somatic mutation in the gene	NGS panel or WES	yes, no, missing	YES	
somatic mutation	*EZH2*	genomic	binary	any identified, filtered somatic mutation in the gene	NGS panel or WES	yes, no, missing	YES	
somatic mutation	*FAM46C*	genomic	binary	any identified, filtered somatic mutation in the gene	NGS panel or WES	yes, no, missing	YES	
somatic mutation	*FAM5GA*	genomic	binary	any identified, filtered somatic mutation in the gene	NGS panel or WES	yes, no, missing	YES	
somatic mutation	*FAS*	genomic	binary	any identified, filtered somatic mutation in the gene	NGS panel or WES	yes, no, missing	YES	
somatic mutation	*FAT1*	genomic	binary	any identified, filtered somatic mutation in the gene	NGS panel or WES	yes, no, missing		
somatic mutation	*FAT4*	genomic	binary	any identified, filtered somatic mutation in the gene	NGS panel or WES	yes, no, missing		
somatic mutation	*FBXW7*	genomic	binary	any identified, filtered somatic mutation in the gene	NGS panel or WES	yes, no, missing		
somatic mutation	*FGFR3*	genomic	binary	any identified, filtered somatic mutation in the gene	NGS panel or WES	yes, no, missing	YES	
somatic mutation	*FLT3*	genomic	binary	any identified, filtered somatic mutation in the gene	NGS panel or WES	yes, no, missing	YES	
somatic mutation	*FOXO1*	genomic	binary	any identified, filtered somatic mutation in the gene	NGS panel or WES	yes, no, missing	YES	
somatic mutation	*FYN*	genomic	binary	any identified, filtered somatic mutation in the gene	NGS panel or WES	yes, no, missing	YES	
somatic mutation	*GATA1*	genomic	binary	any identified, filtered somatic mutation in the gene	NGS panel or WES	yes, no, missing	YES	
somatic mutation	*GATA2*	genomic	binary	any identified, filtered somatic mutation in the gene	NGS panel or WES	yes, no, missing	YES	
somatic mutation	*GFLI*	genomic	binary	any identified, filtered somatic mutation in the gene	NGS panel or WES	yes, no, missing	YES	
somatic mutation	*GNA13*	genomic	binary	any identified, filtered somatic mutation in the gene	NGS panel or WES	yes, no, missing	YES	
somatic mutation	*GNAS*	genomic	binary	any identified, filtered somatic mutation in the gene	NGS panel or WES	yes, no, missing	YES	
somatic mutation	*GPR183*	genomic	binary	any identified, filtered somatic mutation in the gene	NGS panel or WES	yes, no, missing	YES	
somatic mutation	*HAVCR2*	genomic	binary	any identified, filtered somatic mutation in the gene	NGS panel or WES	yes, no, missing		

somatic mutation	*HI4*	genomic	binary	any identified, filtered somatic mutation in the gene	NGS panel or WES	yes, no, missing	YES	
somatic mutation	*HIST1H1C*	genomic	binary	any identified, filtered somatic mutation in the gene	NGS panel or WES	yes, no, missing	YES	
somatic mutation	*HIST1H1E*	genomic	binary	any identified, filtered somatic mutation in the gene	NGS panel or WES	yes, no, missing	YES	
somatic mutation	*HRAS*	genomic	binary	any identified, filtered somatic mutation in the gene	NGS panel or WES	yes, no, missing	YES	
somatic mutation	*HRNRPK*	genomic	binary	any identified, filtered somatic mutation in the gene	NGS panel or WES	yes, no, missing	YES	
somatic mutation	*HUWE1*	genomic	binary	any identified, filtered somatic mutation in the gene	NGS panel or WES	yes, no, missing	YES	
somatic mutation	*HVCN1*	genomic	binary	any identified, filtered somatic mutation in the gene	NGS panel or WES	yes, no, missing	YES	
somatic mutation	*ID3*	genomic	binary	any identified, filtered somatic mutation in the gene	NGS panel or WES	yes, no, missing	YES	
somatic mutation	*IDH1*	genomic	binary	any identified, filtered somatic mutation in the gene	NGS panel or WES	yes, no, missing	YES	
somatic mutation	*IDH2*	genomic	binary	any identified, filtered somatic mutation in the gene	NGS panel or WES	yes, no, missing	YES	
somatic mutation	*IFNGR1*	genomic	binary	any identified, filtered somatic mutation in the gene	NGS panel or WES	yes, no, missing	YES	
somatic mutation	*IGLL5*	genomic	binary	any identified, filtered somatic mutation in the gene	NGS panel or WES	yes, no, missing		
somatic mutation	*IKZF1*	genomic	binary	any identified, filtered somatic mutation in the gene	NGS panel or WES	yes, no, missing	YES	
somatic mutation	*IKZF3*	genomic	binary	any identified, filtered somatic mutation in the gene	NGS panel or WES	yes, no, missing	YES	
somatic mutation	*IL2RG*	genomic	binary	any identified, filtered somatic mutation in the gene	NGS panel or WES	yes, no, missing	YES	
somatic mutation	*IL7R*	genomic	binary	any identified, filtered somatic mutation in the gene	NGS panel or WES	yes, no, missing	YES	
somatic mutation	*IRAK1*	genomic	binary	any identified, filtered somatic mutation in the gene	NGS panel or WES	yes, no, missing	YES	
somatic mutation	*IRF4*	genomic	binary	any identified, filtered somatic mutation in the gene	NGS panel or WES	yes, no, missing	YES	
somatic mutation	*IRF8*	genomic	binary	any identified, filtered somatic mutation in the gene	NGS panel or WES	yes, no, missing	YES	
somatic mutation	*ITK*	genomic	binary	any identified, filtered somatic mutation in the gene	NGS panel or WES	yes, no, missing	YES	
somatic mutation	*ITPKB*	genomic	binary	any identified, filtered somatic mutation in the gene	NGS panel or WES	yes, no, missing	YES	
somatic mutation	*JAK2*	genomic	binary	any identified, filtered somatic mutation in the gene	NGS panel or WES	yes, no, missing	YES	
somatic mutation	*JAK3*	genomic	binary	any identified, filtered somatic mutation in the gene	NGS panel or WES	yes, no, missing		YES
somatic mutation	*KDM6A*	genomic	binary	any identified, filtered somatic mutation in the gene	NGS panel or WES	yes, no, missing	YES	
somatic mutation	*KIT*	genomic	binary	any identified, filtered somatic mutation in the gene	NGS panel or WES	yes, no, missing	YES	
somatic mutation	*KLF16*	genomic	binary	any identified, filtered somatic mutation in the gene	NGS panel or WES	yes, no, missing	YES	
somatic mutation	*KLF2*	genomic	binary	any identified, filtered somatic mutation in the gene	NGS panel or WES	yes, no, missing	YES	
somatic mutation	*KLHL6*	genomic	binary	any identified, filtered somatic mutation in the gene	NGS panel or WES	yes, no, missing	YES	
somatic mutation	*KMT2A*	genomic	binary	any identified, filtered somatic mutation in the gene	NGS panel or WES	yes, no, missing	YES	
somatic mutation	*KMT2C*	genomic	binary	any identified, filtered somatic mutation in the gene	NGS panel or WES	yes, no, missing		
somatic mutation	*KMT2D*	genomic	binary	any identified, filtered somatic mutation in the gene	NGS panel or WES	yes, no, missing		
somatic mutation	*KRAS*	genomic	binary	any identified, filtered somatic mutation in the gene	NGS panel or WES	yes, no, missing		YES
somatic mutation	*LRP1B*	genomic	binary	any identified, filtered somatic mutation in the gene	NGS panel or WES	yes, no, missing	YES	
somatic mutation	*LTB*	genomic	binary	any identified, filtered somatic mutation in the gene	NGS panel or WES	yes, no, missing	YES	
somatic mutation	*LYN*	genomic	binary	any identified, filtered somatic mutation in the gene	NGS panel or WES	yes, no, missing	YES	
somatic mutation	*MALT1*	genomic	binary	any identified, filtered somatic mutation in the gene	NGS panel or WES	yes, no, missing		
somatic mutation	*MAP2K1*	genomic	binary	any identified, filtered somatic mutation in the gene	NGS panel or WES	yes, no, missing	YES	
somatic mutation	*MAP3K14*	genomic	binary	any identified, filtered somatic mutation in the gene	NGS panel or WES	yes, no, missing	YES	
somatic mutation	*MAPK1*	genomic	binary	any identified, filtered somatic mutation in the gene	NGS panel or WES	yes, no, missing	YES	
somatic mutation	*MAX*	genomic	binary	any identified, filtered somatic mutation in the gene	NGS panel or WES	yes, no, missing	YES	
somatic mutation	*MED12*	genomic	binary	any identified, filtered somatic mutation in the gene	NGS panel or WES	yes, no, missing	YES	
somatic mutation	*MEF2B*	genomic	binary	any identified, filtered somatic mutation in the gene	NGS panel or WES	yes, no, missing		YES

somatic mutation	*MFHAS1*	genomic	binary	any identified, filtered somatic mutation in the gene	NGS panel or WES	yes, no, missing		YES
somatic mutation	*MPL*	genomic	binary	any identified, filtered somatic mutation in the gene	NGS panel or WES	yes, no, missing	YES	
somatic mutation	*MUC2*	genomic	binary	any identified, filtered somatic mutation in the gene	NGS panel or WES	yes, no, missing		
somatic mutation	*MYC*	genomic	binary	any identified, filtered somatic mutation in the gene	NGS panel or WES	yes, no, missing	YES	
somatic mutation	*MYD88*	genomic	binary	any identified, filtered somatic mutation in the gene	NGS panel or WES	yes, no, missing		YES
somatic mutation	*NCOR2*	genomic	binary	any identified, filtered somatic mutation in the gene	NGS panel or WES	yes, no, missing		
somatic mutation	*NF1*	genomic	binary	any identified, filtered somatic mutation in the gene	NGS panel or WES	yes, no, missing		
somatic mutation	*NFKB2*	genomic	binary	any identified, filtered somatic mutation in the gene	NGS panel or WES	yes, no, missing	YES	
somatic mutation	*NFKBIA*	genomic	binary	any identified, filtered somatic mutation in the gene	NGS panel or WES	yes, no, missing	YES	
somatic mutation	*NFKBIE*	genomic	binary	any identified, filtered somatic mutation in the gene	NGS panel or WES	yes, no, missing		
somatic mutation	*NLGN1*	genomic	binary	any identified, filtered somatic mutation in the gene	NGS panel or WES	yes, no, missing		YES
somatic mutation	*NOTCH1*	genomic	binary	any identified, filtered somatic mutation in the gene	NGS panel or WES	yes, no, missing		
somatic mutation	*NOTCH2*	genomic	binary	any identified, filtered somatic mutation in the gene	NGS panel or WES	yes, no, missing		
somatic mutation	*NPM1*	genomic	binary	any identified, filtered somatic mutation in the gene	NGS panel or WES	yes, no, missing	YES	
somatic mutation	*NRAS*	genomic	binary	any identified, filtered somatic mutation in the gene	NGS panel or WES	yes, no, missing	YES	
somatic mutation	*NSD2*	genomic	binary	any identified, filtered somatic mutation in the gene	NGS panel or WES	yes, no, missing		
somatic mutation	*NXFL1*	genomic	binary	any identified, filtered somatic mutation in the gene	NGS panel or WES	yes, no, missing	YES	
somatic mutation	*P2RY8*	genomic	binary	any identified, filtered somatic mutation in the gene	NGS panel or WES	yes, no, missing	YES	
somatic mutation	*PAX5*	genomic	binary	any identified, filtered somatic mutation in the gene	NGS panel or WES	yes, no, missing	YES	
somatic mutation	*PCBP1*	genomic	binary	any identified, filtered somatic mutation in the gene	NGS panel or WES	yes, no, missing	YES	
somatic mutation	*PHF6*	genomic	binary	any identified, filtered somatic mutation in the gene	NGS panel or WES	yes, no, missing	YES	
somatic mutation	*PIGA*	genomic	binary	any identified, filtered somatic mutation in the gene	NGS panel or WES	yes, no, missing	YES	
somatic mutation	*PIK3CA*	genomic	binary	any identified, filtered somatic mutation in the gene	NGS panel or WES	yes, no, missing	YES	
somatic mutation	*PIK3RL*	genomic	binary	any identified, filtered somatic mutation in the gene	NGS panel or WES	yes, no, missing	YES	
somatic mutation	*PIM1*	genomic	binary	any identified, filtered somatic mutation in the gene	NGS panel or WES	yes, no, missing	YES	
somatic mutation	*PKHD1L1*	genomic	binary	any identified, filtered somatic mutation in the gene	NGS panel or WES	yes, no, missing	YES	
somatic mutation	*PLCG1*	genomic	binary	any identified, filtered somatic mutation in the gene	NGS panel or WES	yes, no, missing	YES	
somatic mutation	*PLCG2*	genomic	binary	any identified, filtered somatic mutation in the gene	NGS panel or WES	yes, no, missing	YES	
somatic mutation	*PLEKHA5*	genomic	binary	any identified, filtered somatic mutation in the gene	NGS panel or WES	yes, no, missing	YES	
somatic mutation	*PLEKHG5*	genomic	binary	any identified, filtered somatic mutation in the gene	NGS panel or WES	yes, no, missing	YES	
somatic mutation	*PML*	genomic	binary	any identified, filtered somatic mutation in the gene	NGS panel or WES	yes, no, missing	YES	
somatic mutation	*POLE*	genomic	binary	any identified, filtered somatic mutation in the gene	NGS panel or WES	yes, no, missing	YES	
somatic mutation	*POT1*	genomic	binary	any identified, filtered somatic mutation in the gene	NGS panel or WES	yes, no, missing		
somatic mutation	*PRDM1*	genomic	binary	any identified, filtered somatic mutation in the gene	NGS panel or WES	yes, no, missing	YES	
somatic mutation	*PRKCI*	genomic	binary	any identified, filtered somatic mutation in the gene	NGS panel or WES	yes, no, missing		
somatic mutation	*PRPF40B*	genomic	binary	any identified, filtered somatic mutation in the gene	NGS panel or WES	yes, no, missing	YES	
somatic mutation	*PTPRD*	genomic	binary	any identified, filtered somatic mutation in the gene	NGS panel or WES	yes, no, missing	YES	
somatic mutation	*PTEN*	genomic	binary	any identified, filtered somatic mutation in the gene	NGS panel or WES	yes, no, missing	YES	
somatic mutation	*PTPN1*	genomic	binary	any identified, filtered somatic mutation in the gene	NGS panel or WES	yes, no, missing	YES	
somatic mutation	*RAD21*	genomic	binary	any identified, filtered somatic mutation in the gene	NGS panel or WES	yes, no, missing	YES	
somatic mutation	*RARA*	genomic	binary	any identified, filtered somatic mutation in the gene	NGS panel or WES	yes, no, missing	YES	
somatic mutation	*RASSF1*	genomic	binary	any identified, filtered somatic mutation in the gene	NGS panel or WES	yes, no, missing	YES	

somatic mutation	*RB1*	genomic	binary	any identified, filtered somatic mutation in the gene	NGS panel or WES	yes, no, missing		
somatic mutation	*RBMX*	genomic	binary	any identified, filtered somatic mutation in the gene	NGS panel or WES	yes, no, missing	YES	
somatic mutation	*RFTN1*	genomic	binary	any identified, filtered somatic mutation in the gene	NGS panel or WES	yes, no, missing	YES	
somatic mutation	*RHOA*	genomic	binary	any identified, filtered somatic mutation in the gene	NGS panel or WES	yes, no, missing	YES	
somatic mutation	*RIPK1*	genomic	binary	any identified, filtered somatic mutation in the gene	NGS panel or WES	yes, no, missing	YES	
somatic mutation	*ROS1*	genomic	binary	any identified, filtered somatic mutation in the gene	NGS panel or WES	yes, no, missing		
somatic mutation	*RPS15*	genomic	binary	any identified, filtered somatic mutation in the gene	NGS panel or WES	yes, no, missing	YES	
somatic mutation	*RRAGC*	genomic	binary	any identified, filtered somatic mutation in the gene	NGS panel or WES	yes, no, missing	YES	
somatic mutation	*RRAS*	genomic	binary	any identified, filtered somatic mutation in the gene	NGS panel or WES	yes, no, missing	YES	
somatic mutation	*RUNX1*	genomic	binary	any identified, filtered somatic mutation in the gene	NGS panel or WES	yes, no, missing	YES	
somatic mutation	*S1PR1*	genomic	binary	any identified, filtered somatic mutation in the gene	NGS panel or WES	yes, no, missing		YES
somatic mutation	*S1PR2*	genomic	binary	any identified, filtered somatic mutation in the gene	NGS panel or WES	yes, no, missing	YES	
somatic mutation	*SAMHD1*	genomic	binary	any identified, filtered somatic mutation in the gene	NGS panel or WES	yes, no, missing		
somatic mutation	*SET2D*	genomic	binary	any identified, filtered somatic mutation in the gene	NGS panel or WES	yes, no, missing		
somatic mutation	*SETB1*	genomic	binary	any identified, filtered somatic mutation in the gene	NGS panel or WES	yes, no, missing	YES	
somatic mutation	*SF1*	genomic	binary	any identified, filtered somatic mutation in the gene	NGS panel or WES	yes, no, missing	YES	
somatic mutation	*SF3A1*	genomic	binary	any identified, filtered somatic mutation in the gene	NGS panel or WES	yes, no, missing	YES	
somatic mutation	*SF3B1*	genomic	binary	any identified, filtered somatic mutation in the gene	NGS panel or WES	yes, no, missing		YES
somatic mutation	*SH2B3*	genomic	binary	any identified, filtered somatic mutation in the gene	NGS panel or WES	yes, no, missing	YES	
somatic mutation	*SLPR2*	genomic	binary	any identified, filtered somatic mutation in the gene	NGS panel or WES	yes, no, missing	YES	
somatic mutation	*SMARCA4*	genomic	binary	any identified, filtered somatic mutation in the gene	NGS panel or WES	yes, no, missing		
somatic mutation	*SMC1A*	genomic	binary	any identified, filtered somatic mutation in the gene	NGS panel or WES	yes, no, missing	YES	
somatic mutation	*SMC3*	genomic	binary	any identified, filtered somatic mutation in the gene	NGS panel or WES	yes, no, missing	YES	
somatic mutation	*SMO*	genomic	binary	any identified, filtered somatic mutation in the gene	NGS panel or WES	yes, no, missing	YES	
somatic mutation	*SOCS1*	genomic	binary	any identified, filtered somatic mutation in the gene	NGS panel or WES	yes, no, missing	YES	
somatic mutation	*SOX11*	genomic	binary	any identified, filtered somatic mutation in the gene	NGS panel or WES	yes, no, missing	YES	
somatic mutation	*SP140*	genomic	binary	any identified, filtered somatic mutation in the gene	NGS panel or WES	yes, no, missing		
somatic mutation	*SPEN*	genomic	binary	any identified, filtered somatic mutation in the gene	NGS panel or WES	yes, no, missing		
somatic mutation	*SRF2*	genomic	binary	any identified, filtered somatic mutation in the gene	NGS panel or WES	yes, no, missing	YES	
somatic mutation	*SRSF2*	genomic	binary	any identified, filtered somatic mutation in the gene	NGS panel or WES	yes, no, missing	YES	
somatic mutation	*STAB1*	genomic	binary	any identified, filtered somatic mutation in the gene	NGS panel or WES	yes, no, missing	YES	
somatic mutation	*STAG1*	genomic	binary	any identified, filtered somatic mutation in the gene	NGS panel or WES	yes, no, missing	YES	
somatic mutation	*STAG2*	genomic	binary	any identified, filtered somatic mutation in the gene	NGS panel or WES	yes, no, missing	YES	
somatic mutation	*STAT3*	genomic	binary	any identified, filtered somatic mutation in the gene	NGS panel or WES	yes, no, missing	YES	
somatic mutation	*STAT5A*	genomic	binary	any identified, filtered somatic mutation in the gene	NGS panel or WES	yes, no, missing	YES	
somatic mutation	*STAT5B*	genomic	binary	any identified, filtered somatic mutation in the gene	NGS panel or WES	yes, no, missing	YES	
somatic mutation	*STAT6*	genomic	binary	any identified, filtered somatic mutation in the gene	NGS panel or WES	yes, no, missing	YES	
somatic mutation	*STK11*	genomic	binary	any identified, filtered somatic mutation in the gene	NGS panel or WES	yes, no, missing	YES	
somatic mutation	*SUZ12*	genomic	binary	any identified, filtered somatic mutation in the gene	NGS panel or WES	yes, no, missing	YES	
somatic mutation	*SVEP1*	genomic	binary	any identified, filtered somatic mutation in the gene	NGS panel or WES	yes, no, missing		YES
somatic mutation	*SYK*	genomic	binary	any identified, filtered somatic mutation in the gene	NGS panel or WES	yes, no, missing	YES	
somatic mutation	*SZT2*	genomic	binary	any identified, filtered somatic mutation in the gene	NGS panel or WES	yes, no, missing		YES

somatic mutation	*TCF3*	genomic	binary	any identified, filtered somatic mutation in the gene	NGS panel or WES	yes, no, missing	YES	
somatic mutation	*TELLXR*	genomic	binary	any identified, filtered somatic mutation in the gene	NGS panel or WES	yes, no, missing	YES	
somatic mutation	*TELLXR1*	genomic	binary	any identified, filtered somatic mutation in the gene	NGS panel or WES	yes, no, missing	YES	
somatic mutation	*TERS*	genomic	binary	any identified, filtered somatic mutation in the gene	NGS panel or WES	yes, no, missing	YES	
somatic mutation	*TERT*	genomic	binary	any identified, filtered somatic mutation in the gene	NGS panel or WES	yes, no, missing	YES	
somatic mutation	*TET2*	genomic	binary	any identified, filtered somatic mutation in the gene	NGS panel or WES	yes, no, missing		YES
somatic mutation	*TF3*	genomic	binary	any identified, filtered somatic mutation in the gene	NGS panel or WES	yes, no, missing	YES	
somatic mutation	*TGM7*	genomic	binary	any identified, filtered somatic mutation in the gene	NGS panel or WES	yes, no, missing	YES	
somatic mutation	*TM2M30A*	genomic	binary	any identified, filtered somatic mutation in the gene	NGS panel or WES	yes, no, missing	YES	
somatic mutation	*TNFAIP3*	genomic	binary	any identified, filtered somatic mutation in the gene	NGS panel or WES	yes, no, missing	YES	
somatic mutation	*TNFRS14*	genomic	binary	any identified, filtered somatic mutation in the gene	NGS panel or WES	yes, no, missing	YES	
somatic mutation	*TP53*	genomic	binary	any identified, filtered somatic mutation in the gene	NGS panel or WES	yes, no, missing		
somatic mutation	*TRAF2*	genomic	binary	any identified, filtered somatic mutation in the gene	NGS panel or WES	yes, no, missing		
somatic mutation	*TRAF3*	genomic	binary	any identified, filtered somatic mutation in the gene	NGS panel or WES	yes, no, missing	YES	
somatic mutation	*TRAF6*	genomic	binary	any identified, filtered somatic mutation in the gene	NGS panel or WES	yes, no, missing	YES	
somatic mutation	*U2AF*	genomic	binary	any identified, filtered somatic mutation in the gene	NGS panel or WES	yes, no, missing	YES	
somatic mutation	*U2AF1*	genomic	binary	any identified, filtered somatic mutation in the gene	NGS panel or WES	yes, no, missing	YES	
somatic mutation	*U2AF2*	genomic	binary	any identified, filtered somatic mutation in the gene	NGS panel or WES	yes, no, missing	YES	
somatic mutation	*UBR5*	genomic	binary	any identified, filtered somatic mutation in the gene	NGS panel or WES	yes, no, missing		
somatic mutation	*VAV1*	genomic	binary	any identified, filtered somatic mutation in the gene	NGS panel or WES	yes, no, missing	YES	
somatic mutation	*WNK1*	genomic	binary	any identified, filtered somatic mutation in the gene	NGS panel or WES	yes, no, missing		YES
somatic mutation	*WT1*	genomic	binary	any identified, filtered somatic mutation in the gene	NGS panel or WES	yes, no, missing	YES	
somatic mutation	*XPO1*	genomic	binary	any identified, filtered somatic mutation in the gene	NGS panel or WES	yes, no, missing	YES	
somatic mutation	*ZFAT*	genomic	binary	any identified, filtered somatic mutation in the gene	NGS panel or WES	yes, no, missing	YES	
somatic mutation	*ZMYM3*	genomic	binary	any identified, filtered somatic mutation in the gene	NGS panel or WES	yes, no, missing	YES	
somatic mutation	*ZRSR2*	genomic	binary	any identified, filtered somatic mutation in the gene	NGS panel or WES	yes, no, missing	YES	
Combined mutation	*TP53 and ATM*	genomic	binary	any identified, filtered somatic mutation in the gene	NGS panel or WES	yes, no, missing		
IGH Hypermutation	IGH locus	genomic	continous		PCR			
Total genes with mutations	any "total_genes"	genomic	continous		NGS panel or WES - engineered			
Total germline mutations	any "total_germline"	genomic	continous	Identified as germline in CLIA NGS panel	NGS panel or WES - engineered			
Clonal Hematopoeisis Score	*TET2, ASXl1, TP53, ATM, CHEK2, JAK2, IDH1*	genomic	continous	From identified germline mutations	NGS panel or WES - engineered	0-7		

Feature	Abbreviation
MIPI	mipi_calc
Biological MIPI	biol_mipi
Interval from treatment to relapse or progression	
Interval from treatment to relapse, progression or death	
Interval from diagnosis to relapse or progression	
Interval from diagnosis to death	
