# Supplementary material for: Integrative Prognostic Machine Learning Models in Mantle Cell Lymphoma
Source: Cancer Res Commun. 2023 Aug 2;3(8):1435–46. doi: 10.1158/2767-9764.CRC-23-0083 (PMC10395375; doi:10.1158/2767-9764.CRC-23-0083)
Supplement: Supplementary Table 3 — Missingness among Full Dataset Features [file crc-23-0083-s04.pdf]

Supplementary Table 3

Missingness Among Full Dataset Features				
Feature	Missing	Complete	Percent Complete	Percent Missing
abnormal_karyotype	177	617	77.71	22.29
add_1	177	617	77.71	22.29
add_10	177	617	77.71	22.29
add_11	176	618	77.83	22.17
add_12	177	617	77.71	22.29
add_13	177	617	77.71	22.29
add_14	177	617	77.71	22.29
add_15	177	617	77.71	22.29
add_16	177	617	77.71	22.29
add_17	177	617	77.71	22.29
add_18	177	617	77.71	22.29
add_19	177	617	77.71	22.29
add_2	177	617	77.71	22.29
add_21	177	617	77.71	22.29
add_22	177	617	77.71	22.29
add_3	176	618	77.83	22.17
add_4	177	617	77.71	22.29
add_5	177	617	77.71	22.29
add_6	177	617	77.71	22.29
add_7	176	618	77.83	22.17
add_8	177	617	77.71	22.29
add_9	177	617	77.71	22.29
age_dx_years	0	794	100.00	0.00
alcohol	39	755	95.09	4.91
arid1a	570	224	28.21	71.79

Missingness Among Full Dataset Features

asxl1	564	230	28.97	71.03
atm	483	311	39.17	60.83
b_symptoms	279	515	64.86	35.14
b2m	314	480	60.45	39.55
bcor	564	230	28.97	71.03
birc3	485	309	38.92	61.08
bm	110	684	86.15	13.85
bmi	440	354	44.58	55.42
btk	485	309	38.92	61.08
bulky	0	794	100.00	0.00
card11	485	309	38.92	61.08
ccnd1	570	224	28.21	71.79
cd79b	485	309	38.92	61.08
celsr3	707	87	10.96	89.04
ch_score	566	228	28.72	71.28
chrom_1_other	177	617	77.71	22.29
chrom_11_other	177	617	77.71	22.29
chrom_13_other	177	617	77.71	22.29
chrom_14_other	177	617	77.71	22.29
chrom_15_other	177	617	77.71	22.29
chrom_17_other	177	617	77.71	22.29
chrom_2_other	177	617	77.71	22.29
chrom_22_other	176	618	77.83	22.17
chrom_3_other	177	617	77.71	22.29
chrom_4_other	177	617	77.71	22.29
chrom_6_other	177	617	77.71	22.29

Missingness Among Full Dataset Features

chrom_8_other	177	617	77.71	22.29
chrom_9_other	177	617	77.71	22.29
chrom_sex_other	176	618	77.83	22.17
complex_kar	176	618	77.83	22.17
cxcr4	485	309	38.92	61.08
del_1	177	617	77.71	22.29
del_10	177	617	77.71	22.29
del_11	175	619	77.96	22.04
del_12	177	617	77.71	22.29
del_13	177	617	77.71	22.29
del_14	177	617	77.71	22.29
del_15	177	617	77.71	22.29
del_16	177	617	77.71	22.29
del_17	177	617	77.71	22.29
del_18	177	617	77.71	22.29
del_2	177	617	77.71	22.29
del_20	176	618	77.83	22.17
del_21	177	617	77.71	22.29
del_22	177	617	77.71	22.29
del_3	177	617	77.71	22.29
del_4	177	617	77.71	22.29
del_6	177	617	77.71	22.29
del_7	177	617	77.71	22.29
del_8	177	617	77.71	22.29
del_9	176	618	77.83	22.17
del_x	177	617	77.71	22.29

Missingness Among Full Dataset Features				
deletion_y	176	618	77.83	22.17
dnmt3a	561	233	29.35	70.65
ecog	389	405	51.01	48.99
egfr	705	89	11.21	88.79
ep300	570	224	28.21	71.79
fat1	485	309	38.92	61.08
gi_involve	432	362	45.59	54.41
hgb	92	702	88.41	11.59
hypermut	700	94	11.84	88.16
igll5	570	224	28.21	71.79
ki_67	142	652	82.12	17.88
kmt2c	707	87	10.96	89.04
kmt2d	570	224	28.21	71.79
ldh	198	596	75.06	24.94
malt1	707	87	10.96	89.04
mef2b	570	224	28.21	71.79
morph	202	592	74.56	25.44
muc2	621	173	21.79	78.21
ncor2	707	87	10.96	89.04
nf1	564	230	28.97	71.03
nfkbie	570	224	28.21	71.79
notch1	477	317	39.92	60.08
notch2	570	224	28.21	71.79
nsd2	570	224	28.21	71.79
pattern	507	287	36.15	63.85
platelets	96	698	87.91	12.09

Missingness Among Full Dataset Features

prior_cancer	20	774	97.48	2.52
prior_chemo	21	773	97.36	2.64
prior_radiation	22	772	97.23	2.77
prkci	707	87	10.96	89.04
pt_id	0	794	100.00	0.00
race	0	794	100.00	0.00
rb1	568	226	28.46	71.54
ros1	707	87	10.96	89.04
s1pr1	570	224	28.21	71.79
samhd1	570	224	28.21	71.79
sex	0	794	100.00	0.00
site	98	696	87.66	12.34
smarca4	570	224	28.21	71.79
smoke	21	773	97.36	2.64
smokeless	27	767	96.60	3.40
sp140	570	224	28.21	71.79
spen	485	309	38.92	61.08
status	0	794	100.00	0.00
t_11_14	175	619	77.96	22.04
t_14_18	177	617	77.71	22.29
tet2	564	230	28.97	71.03
tetraploid_population	177	617	77.71	22.29
total_genes_mutated	474	320	40.30	59.70
total_germ_variants	566	228	28.72	71.28
tp53	476	318	40.05	59.95
traf2	485	309	38.92	61.08

Missingness Among Full Dataset Features				
ubr5	570	224	28.21	71.79
wbc	54	740	93.20	6.80
