# Supplementary material for: Integrative Prognostic Machine Learning Models in Mantle Cell Lymphoma
Source: Cancer Res Commun. 2023 Aug 2;3(8):1435–46. doi: 10.1158/2767-9764.CRC-23-0083 (PMC10395375; doi:10.1158/2767-9764.CRC-23-0083)
Supplement: Supplementary Table 4 — Hyperparameters for XGBoost Models [file crc-23-0083-s05.pdf]

Supplementary Table 4: Hyperparameters fo XGBoost Models

Model	Mtry	Min. n	Tree Depth	Learn Rate	Loss Reduction	Sample Size	Trees	Set Name
**all features**	129	3	11	0.007838	0.00000073	0.2023401	2000	Model 25
**clinical only**	13	6	15	0.00212333	3.35E-02	0.6327761	2000	Model 11
**clinical + cyto**	44	6	15	0.00212329	3.35E-02	0.6327761	2000	Model 11
**clinical + ngs**	35	6	15	0.00212329	0.03347573	0.6327761	2000	Model 11
**cyto only**	39	5	2	0.00042332	2.28666E-07	0.8644	2000	Model 36
**ngs only**	23	6	15	0.0021233	0.00334757	0.6328	2000	Model 11
**parsimonious**	11	6	15	0.00212329	0.03347573	0.6328	2000	Model 11
