# Supplementary material for: Integrative Prognostic Machine Learning Models in Mantle Cell Lymphoma
Source: Cancer Res Commun. 2023 Aug 2;3(8):1435–46. doi: 10.1158/2767-9764.CRC-23-0083 (PMC10395375; doi:10.1158/2767-9764.CRC-23-0083)
Supplement: Supplementary Table 5 — Coefficients from the Multivariate Generalized Linear Model [file crc-23-0083-s06.pdf]

# Supplementary Table 5: Multivariate Model (GLM)

| Supplementary Table 5: Multivariate Model (GLM) | | | | | |
|---|---|---|---|---|---|
| **Feature** | **Coefficient** | **Odds Ratio** | **Std. Error** | **Statistic** | **p-value** |
| Ki 67 % | 0.67 | 1.95 | 0.14 | 4.84 | 0.00 |
| Lactase Dehydrogenase | 0.89 | 2.44 | 0.21 | 4.33 | 0.00 |
| Age at Diagnosis | 0.27 | 1.31 | 0.11 | 2.40 | 0.02 |
| Leukemic variant | −2.28 | 0.10 | 0.99 | −2.30 | 0.02 |
| Never Smoker | −0.49 | 0.61 | 0.22 | −2.24 | 0.03 |
| Number of Somatic Mutations | 0.23 | 1.26 | 0.11 | 2.17 | 0.03 |
| Bone Marrow Involvement % | 0.27 | 1.31 | 0.13 | 2.06 | 0.04 |
| White blood cell count | 0.40 | 1.49 | 0.20 | 1.96 | 0.05 |
| Beta 2 Microglobulin | −0.24 | 0.79 | 0.12 | −1.94 | 0.05 |
| B-symptoms | 0.43 | 1.54 | 0.26 | 1.69 | 0.09 |
| Blastoid Morphology | −0.95 | 0.39 | 0.61 | −1.55 | 0.12 |
| Hemoglobin | −0.17 | 0.84 | 0.12 | −1.39 | 0.17 |
| *NOTCH1* mutation | −1.00 | 0.37 | 0.75 | −1.33 | 0.18 |
| Platelets | −0.13 | 0.88 | 0.11 | −1.25 | 0.21 |
| GI Involvement | 0.30 | 1.35 | 0.26 | 1.14 | 0.25 |
| *BIRC3* mutation | −0.41 | 0.66 | 0.44 | −0.93 | 0.35 |
| *ROS1* mutation | 0.51 | 1.67 | 0.55 | 0.93 | 0.35 |
| ECOG Status 2 | −0.92 | 0.40 | 1.05 | −0.88 | 0.38 |
| Blastoid and Pleomorphic Morphology | 1.11 | 3.03 | 1.38 | 0.80 | 0.42 |
| ECOG Status 1 | −0.21 | 0.81 | 0.29 | −0.74 | 0.46 |
| Classic Morphology | −0.35 | 0.70 | 0.56 | −0.63 | 0.53 |
| Pleomorphic Morphology | 0.31 | 1.36 | 0.72 | 0.43 | 0.67 |
| Current Smoker | 0.11 | 1.12 | 0.42 | 0.27 | 0.79 |
| BMI | 0.02 | 1.02 | 0.10 | 0.15 | 0.88 |
| *CARD11* mutation | −0.02 | 0.98 | 0.68 | −0.03 | 0.97 |
| ECOG Status 3 or 4 | 14.39 | 1776223.43 | 597.96 | 0.02 | 0.98 |
| *TP53* mutation | 0.00 | 1.00 | 0.28 | −0.02 | 0.99 |
| (Intercept) | 0.09 | 1.09 | 0.59 | 0.16 | 0.87 |
