# Supplementary material for: Integrative Prognostic Machine Learning Models in Mantle Cell Lymphoma
Source: Cancer Res Commun. 2023 Aug 2;3(8):1435–46. doi: 10.1158/2767-9764.CRC-23-0083 (PMC10395375; doi:10.1158/2767-9764.CRC-23-0083)
Supplement: Supplementary Table 6 — Univariate survival comparisons from features identified from XGBoost. [file crc-23-0083-s07.pdf]

Supplementary Table 6: Statistics from Log-Rank Test of differences in Overall Survival (OS) and Progression-free Survival (PFS) in patient groups.

Feature	**OS**		**PFS**	
	**n**	$\chi^2(df)$	**n**	$\chi^2(df)$
ECOG	405	76 (3)***	372	57.9 (3)***
Morphology	592	52.9 (2)***	544	82.1 (2)***
TP53 Mutation	318	16.3 (1)***	289	25.6 (1)***
B2M	480	12.7 (1)***	441	13.4 (1)***
Age at Diagnosis	794	18.3 (1)***	718	9.5 (1)**
Bone Marrow	684	18.2 (1)***	623	13.1 (1)***
Platelets	698	22.5(1)***	637	24.2 (1)***
WBC	733	12.7 (2)**	670	22.4 (2)***
Ki-67%	580	10.5 (1)**	530	33.3 (1)***
BMI	440	7.4 (1)**	317	3.0 (1)
Smoking Status	773	9.4 (2)**	701	12.4 (2)**
LDH	596	4.8 (1)*	538	5.1 (1)*
Mutation Count	320	0.0 (1)	291	0.9 (1)

ECOG = Eastern Cooperative Oncology Group performance status; B2M = Beta 2 microglobulin category; WBC = white blood cell count category; BMI = body-mass index; LDH = lactase dehydrogenase level group.
df = degrees of freedom
n = number of patients with data for the identified feature.
P value: * <0.05, ** <0.01, ***<0.001
