# Supplementary material for: Integrative Prognostic Machine Learning Models in Mantle Cell Lymphoma
Source: Cancer Res Commun. 2023 Aug 2;3(8):1435–46. doi: 10.1158/2767-9764.CRC-23-0083 (PMC10395375; doi:10.1158/2767-9764.CRC-23-0083)

Supplementary Figure 2: Variable Importance from Full XGBoost Model (training cross-validation sets)

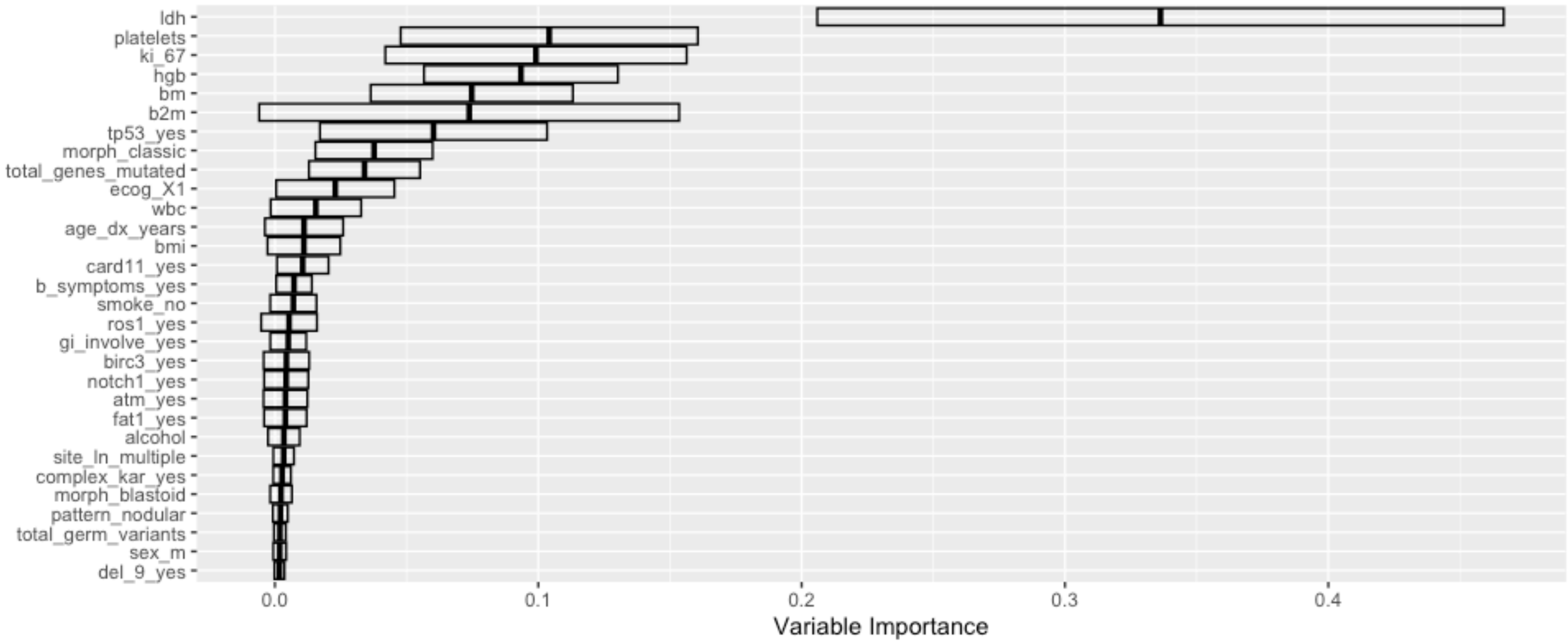

Supplement: Supplementary Figure 2 — S2. Variable Importance from Full XGBoost Model (training cross-validation sets) [file crc-23-0083-s09.pdf]
