# Supplementary figures and images for: Integrative Prognostic Machine Learning Models in Mantle Cell Lymphoma
Source: Cancer Res Commun. 2023 Aug 2;3(8):1435–46. doi: 10.1158/2767-9764.CRC-23-0083 (PMC10395375; doi:10.1158/2767-9764.CRC-23-0083)

A

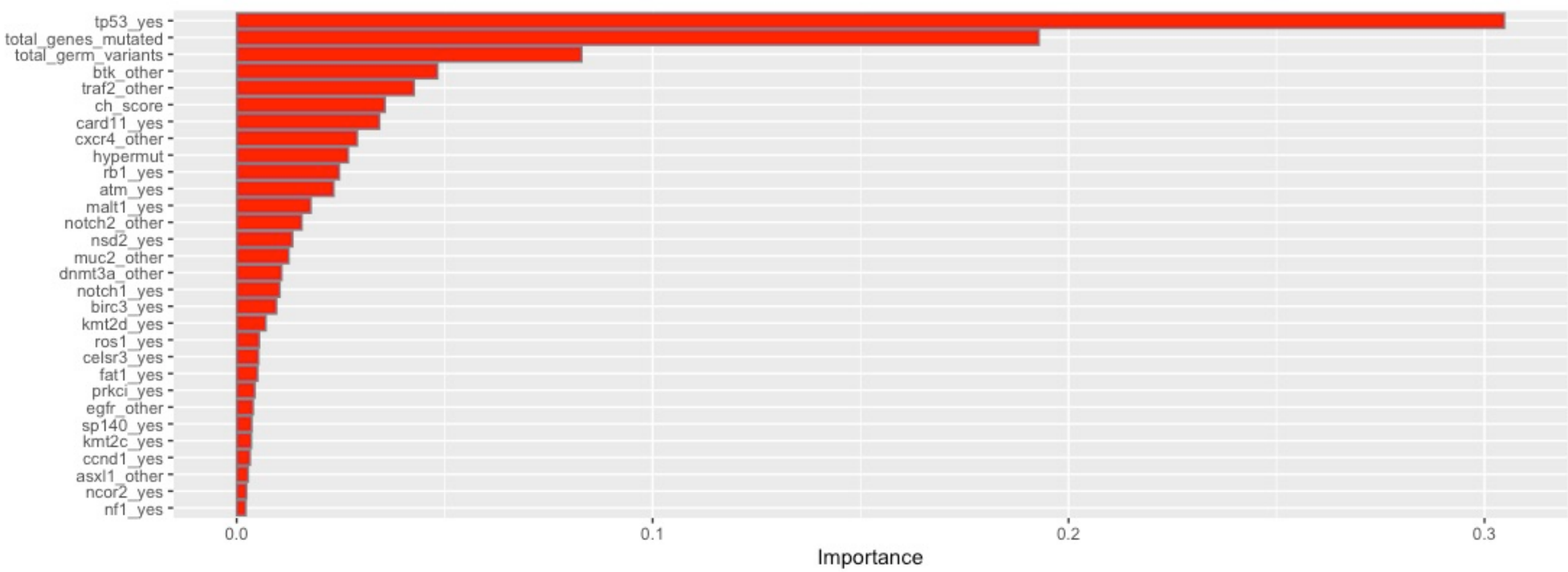

B

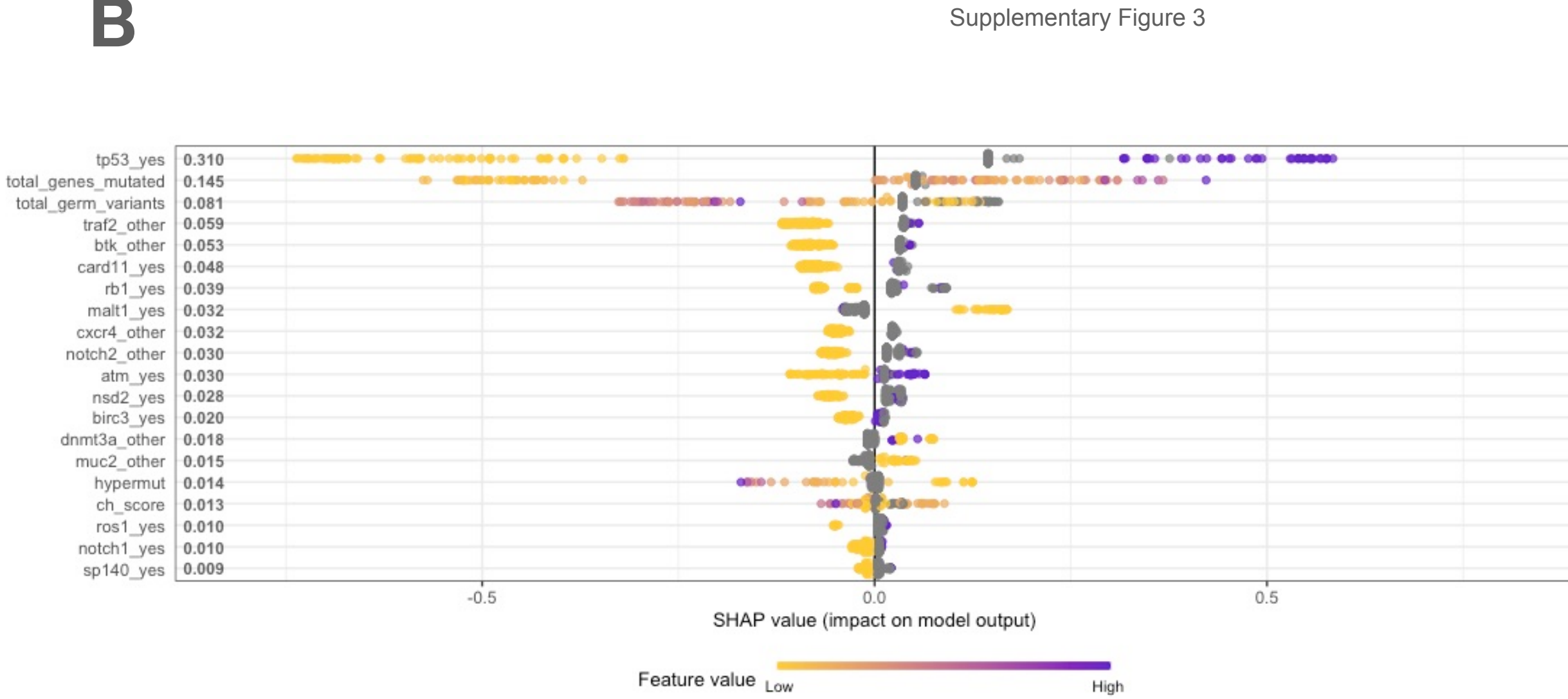

C

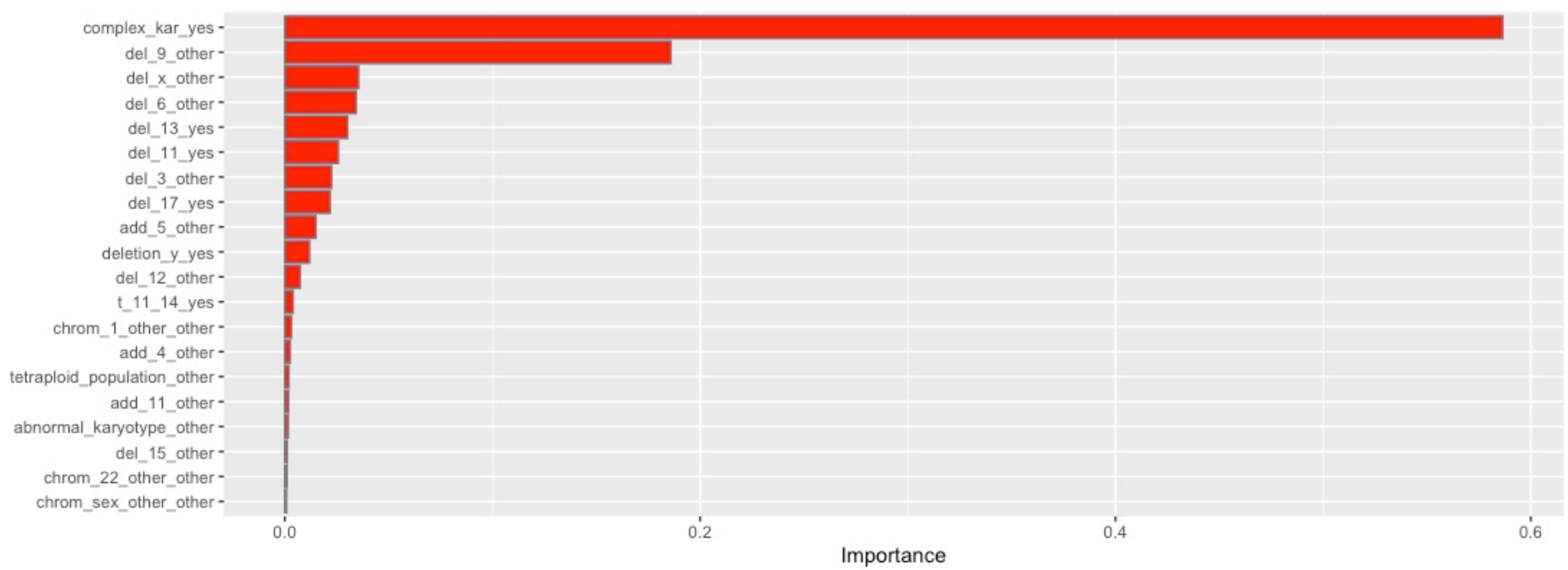

D

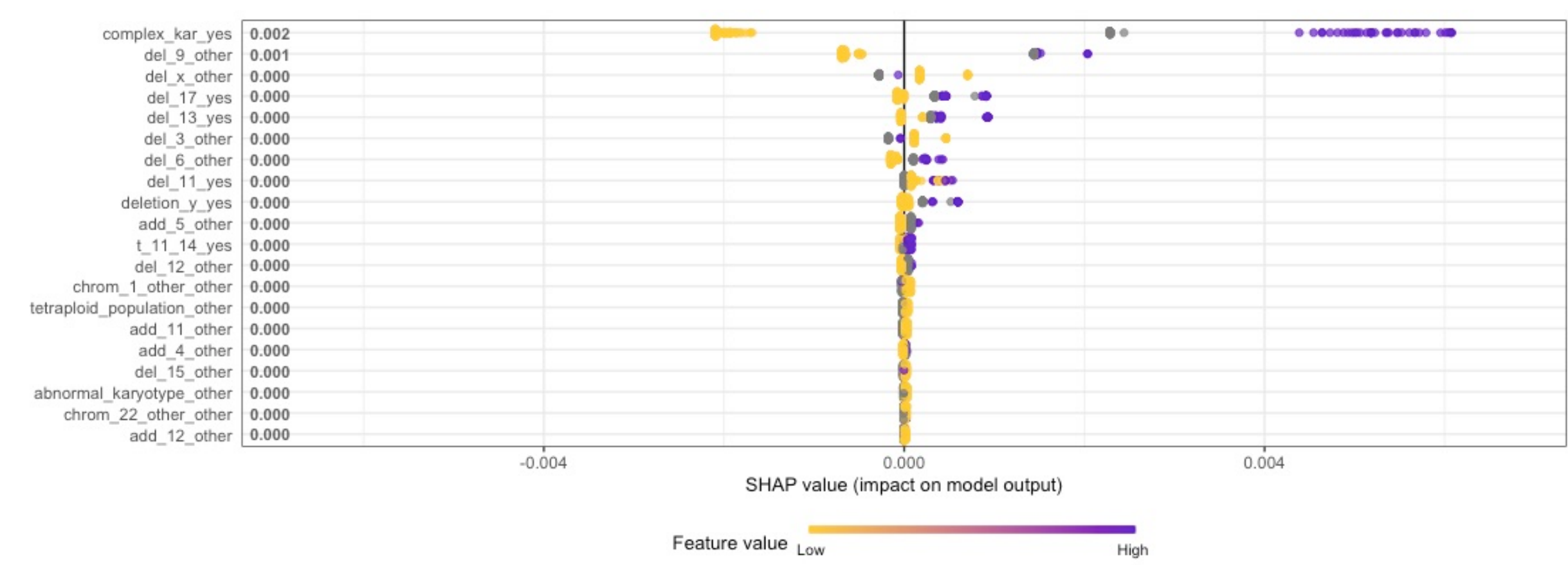

E

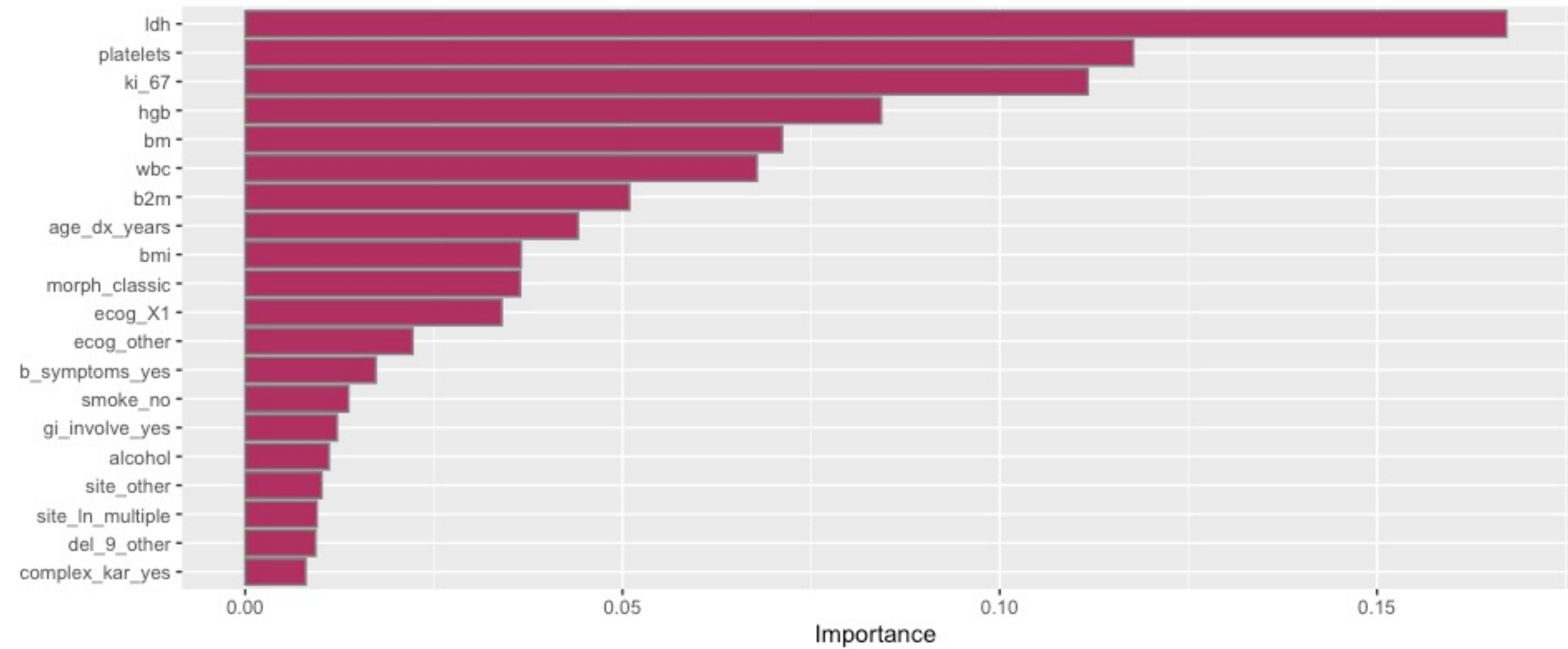

F

Supplementary Figure 3

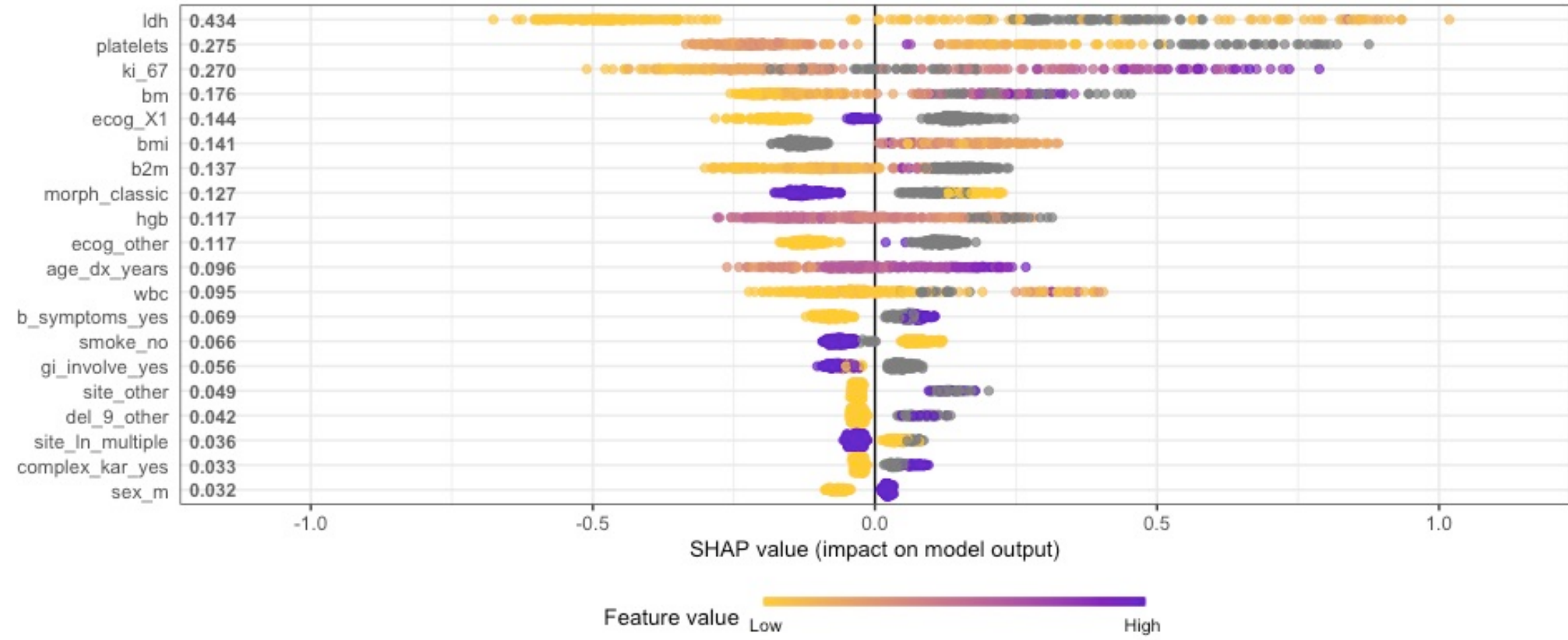

G

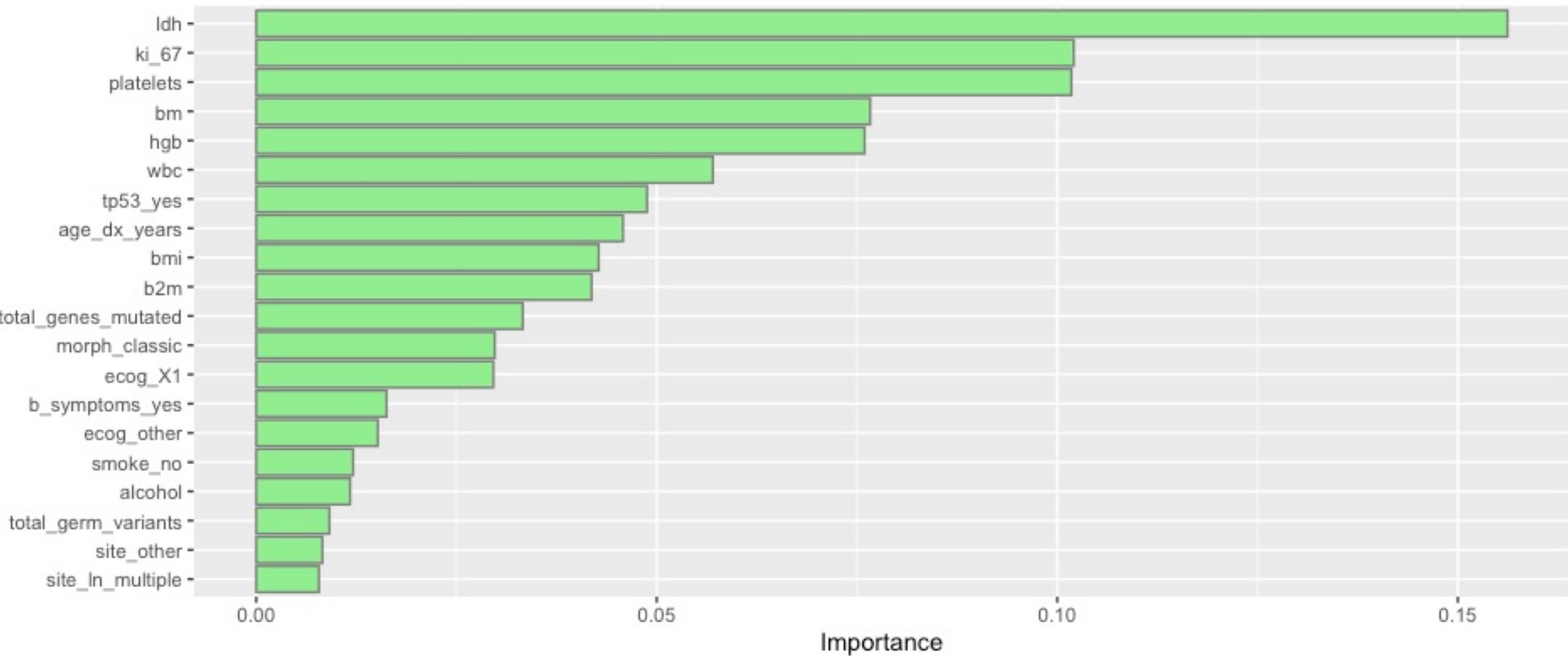

H

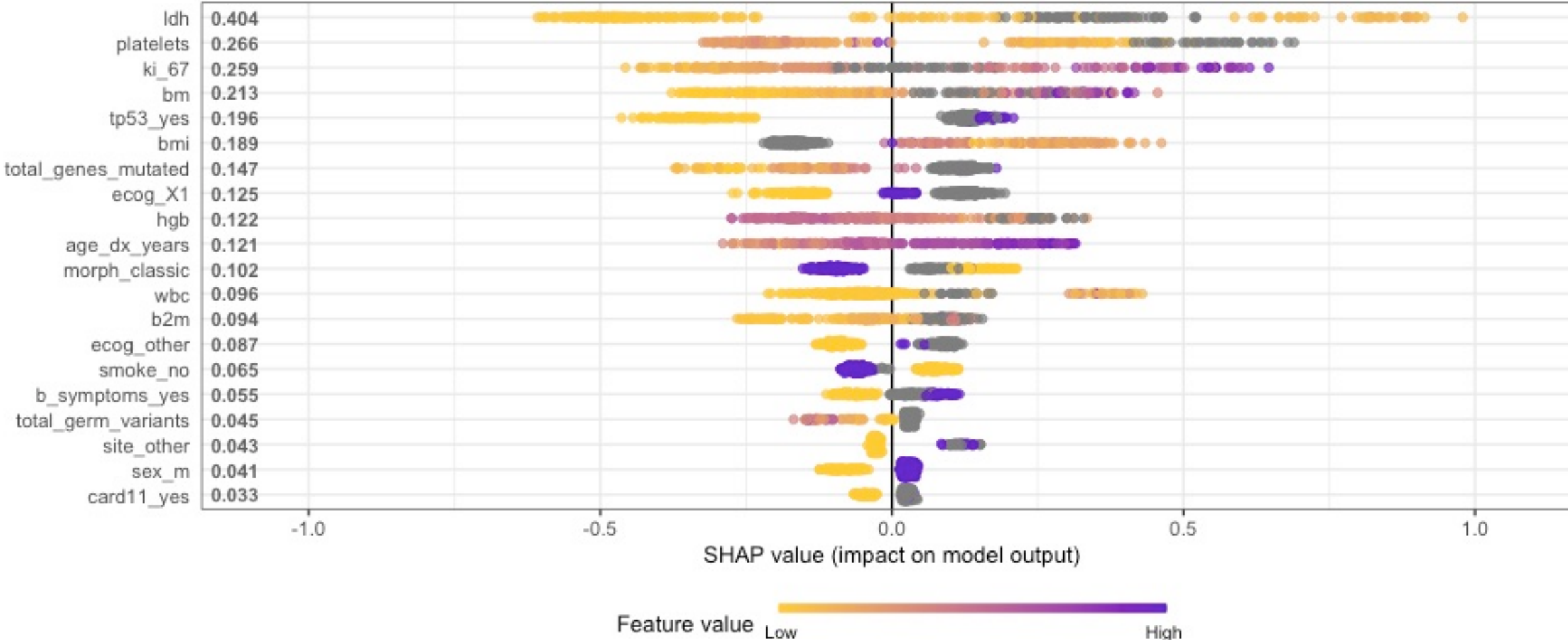

I

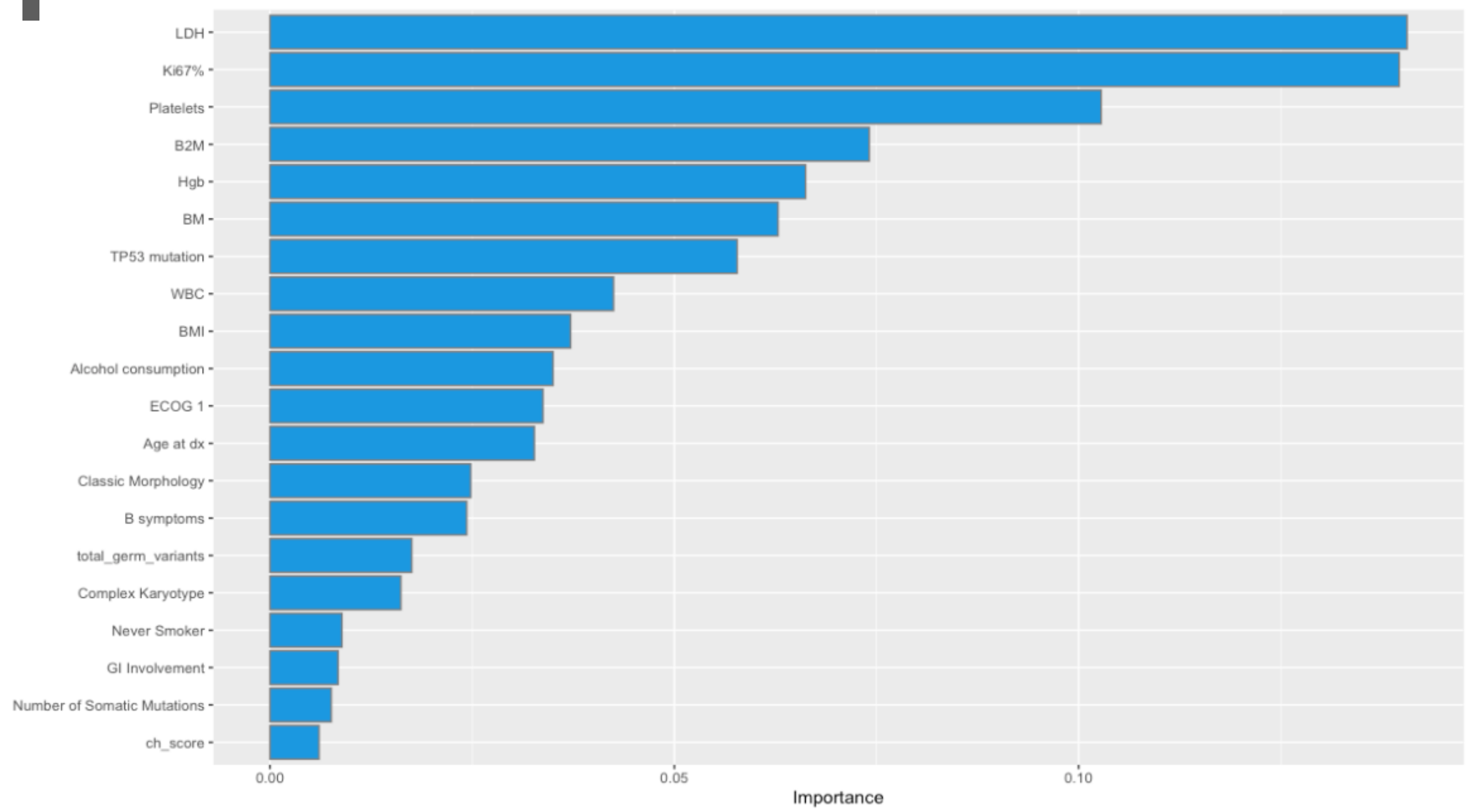

J

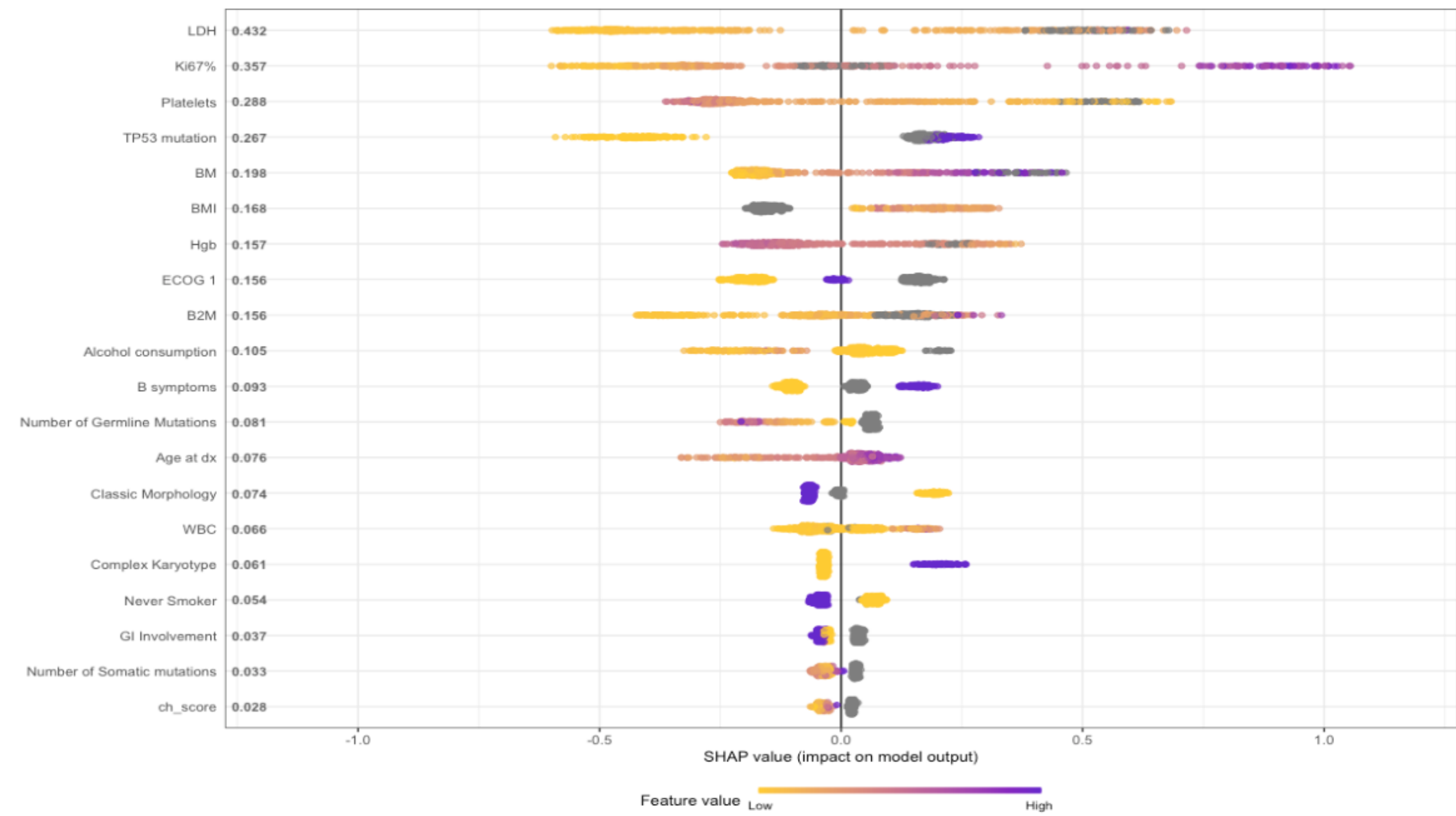

Supplement: Supplementary Figure 3 — S3. Feature Importance from other XGBoost models [file crc-23-0083-s10.pdf]

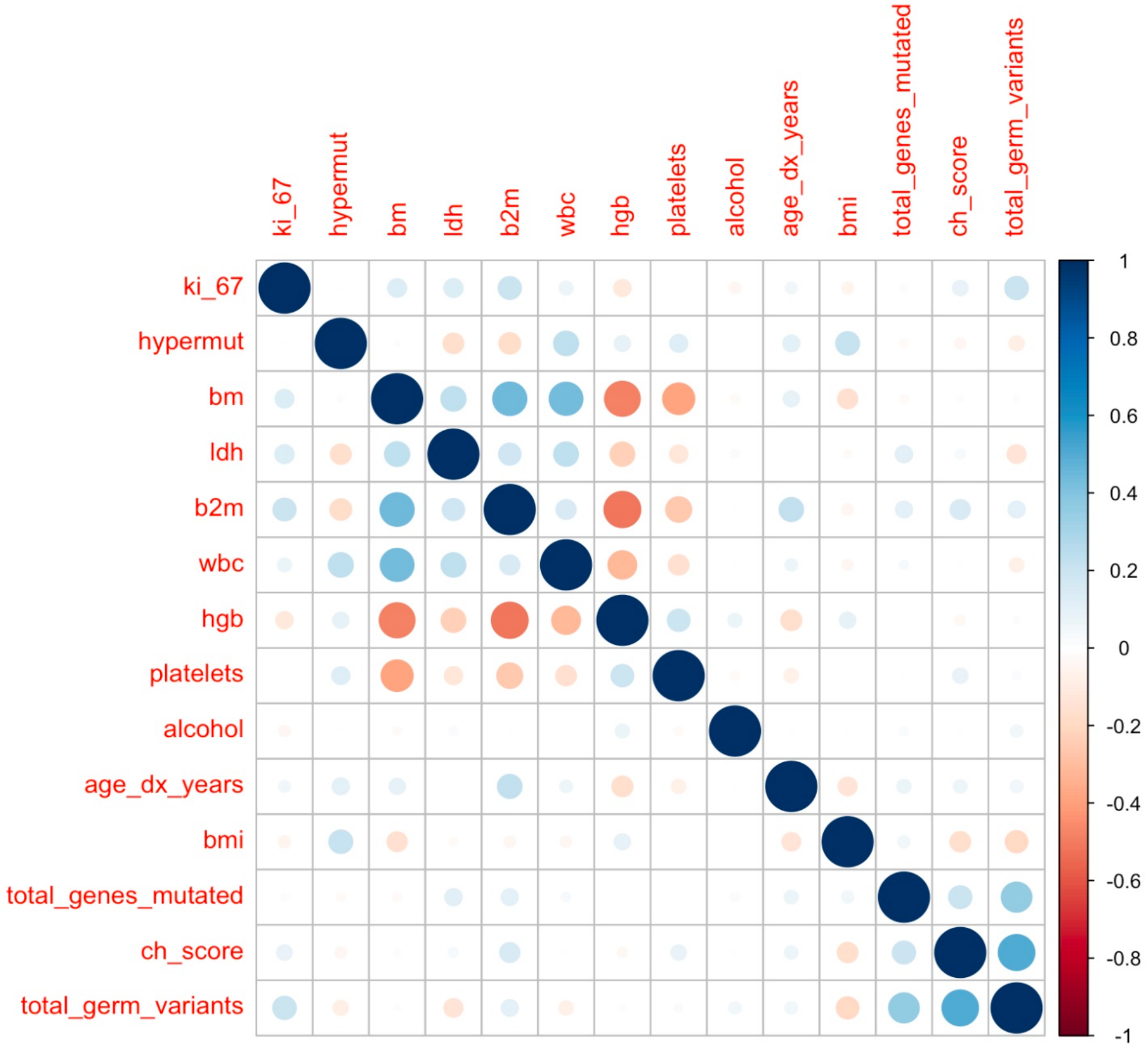

Supplement: Supplementary Figure 4 — S4. Correlation Plot of Numeric Features [file crc-23-0083-s11.pdf]
