# Supplementary material for: Integrative Prognostic Machine Learning Models in Mantle Cell Lymphoma
Source: Cancer Res Commun. 2023 Aug 2;3(8):1435–46. doi: 10.1158/2767-9764.CRC-23-0083 (PMC10395375; doi:10.1158/2767-9764.CRC-23-0083)
Supplement: Supplementary Methods — Supplementary detailed methods [file crc-23-0083-s12.pdf]

EXTENDED METHODS

Patient Database Curation

Patient data were extracted from electronic medical records (EMR) using approved methods from our institutional review boards (IRB). The samples used for genetic sequencing were collected using our approved IRB laboratory protocol for tissue banking and with patient consent.

All predictive features were obtained from baseline or pre-treatment data collection. No patients were excluded due to missing data, as they were imputed by the XGBoost algorithm or by KNN imputation.

Ki-67% was assessed by counting stained cells in either a lymph node or bone marrow sample taken at baseline (positively stained cells/total number of tumor cells). The highest recorded ki-67%, was used for the analysis if multiple samples were procured. Similarly, bone marrow infiltration (BM) was qualitatively measured by assessing the number of malignant cells in the BM samples. If more than one BM sample was obtained at baseline, the highest percentage was used for the analysis. Lactase dehydrogenase (LDH) levels were measured and compared against our laboratory's upper limit of normal (ULN), 225 U/L. We were limited by our lack of knowledge of the ULN of external laboratories that submitted outside records.

Assessments and Endpoints

Patients who relapsed, progressed, or died after the first treatment were classified as having aggressive MCL. Patients who died without treatment were also classified as having aggressive MCL. Patients who were not yet treated or remained in remission after treatment were classified as having indolent/responsive MCL. Response and remission evaluation were based on the Response Evaluation Criteria in Lymphoma (RECIL) [1] or the Lugano Classification [2].

PFS was defined from the date of the first treatment to the date of relapse, progression, or death. Patients who were not treated were excluded from this model. OS was defined as the period from the date of diagnosis to the date of death; all patients were included in this model.

Categorical Variable Creation for Survival Comparison Groups

Groups were categorized accordingly:

- Ki-67%
    - > 20 = "high"
    - <= 20 = "low"
- LDH (lactase dehydrogenase) – 255 ULN
    - > = 255 IL/U = "high"
    - < 255 IL/U= normal
- WBC (white blood cell count)
    - > 10,700/μL= "high"
    - between 10,700 and 3700 μL = "normal"
    - < 3700/μL = "low

- Mutation count
    - “Greater than five”
    - “Five or Less”
- Beta 2 microglobulin
    - > 1.8 mg/L = “high”
    - < = 1.8 mg/L= “normal”
- Bone marrow infiltration
    - > 5% = “involved”
    - < = 5% = “minimal or none”
- Platelets
    - > 150 $/\mu L$ = “normal
    - < = 150$/\mu L$ = “low
- BMI
    - > 24.9 = “obese or overweight”
    - < 24.9 = “normal or underweight:=”
- Age (median age of group 63)
    - > = 63 = “over 63 years old at diagnosis”
    - < 63 = “under 63 years old at diagnosis”

Software packages and statistical analyses

XGBoost, GLM, and Cox regression models were constructed using the R programming libraries: TidyModels [3] and survival [4]. The XGBoost algorithm was executed via parallel processing on an

Intel-based MacBook Pro. VIP and SHAP values were visualized using the libraries: SHAP for XGBoost [5] and VIP [6]. The model REST API deployment was performed using the Vetiver and Plumber [7] packages. Other information about the software used for visualization can be found in our GitHub repository.

Cytogenetic Analyses

For general chromosome analysis and assessment of copy number variations relative to ploidy level, metaphases were analyzed using comparative genomic hybridization (CGH) for duplications, deletions, and additions. Where CGH was not available, conventional karyotyping was used to determine large aberrations.

Fluorescent in situ hybridization (FISH) was performed at our institution using dual color probes from Abbott Molecular Inc (Abbott Park, Illinois, USA) to identify translocations and other targets of interest. Interphases were analyzed, and the percentage of cells that were positive for the target was determined. The cytogenetics laboratory has established various expected cutoffs, which are available upon request.

Whole Exome Sequencing (WES)

Complete information about WES can be found in our previously published papers. [8-10] Generally, WES methods conducted on samples from which data were incorporated are as follows:

Sample processing and DNA extraction:

We separated Mononuclear cells by Ficoll-Hypaque density centrifugation. The cells were isolated using anti-CD19 magnetic microbeads (Miltenyi Biotec, Auburn, CA). The isolated tumor cells were maintained in RPMI-1640 (Life Technologies, Grand Island, NY) supplemented with 10% heat-inactivated fetal bovine serum, penicillin (10,000 units/mL, Sigma), streptomycin (10 mg/mL, Sigma), gentamicin (50 mg/mL, Sigma), and L-glutamine (29.2 mg/mL, Life Technologies). For DNA sequencing, fresh specimens were immediately placed into RNALater solution after surgical biopsy or Ficoll-Hypaque density centrifugation and selective CD19 magnetic isolation of $CD19^+$ cells from bone marrow aspiration or peripheral blood.

Whole blood samples processing:

10-20 mL of peripheral blood was collected in EDTA tubes and processed within 3 h of phlebotomy. Tubes were centrifuged at 1,800g for 10 minutes, and plasma was frozen at -80° C in 1-2 mL aliquots. A portion of the leukocyte-enriched plasma-depleted whole blood was frozen at -80° C in 1-2 mL aliquots and used to isolate germline genomic DNA. All procedures were performed in a cold buffer or ice.

Library Preparation:

Indexed libraries were prepared from 500 ng of Biorupter Ultrasonicator (Diagenode, Denville, NJ, USA)-sheared genomic DNA using the KAPA Hyper Library Preparation Kit (KAPABiosystems, Wilmington, MA, USA). Indexed libraries were prepared for capture using six cycles of prelitigation-mediated PCR amplification. Following amplification and reaction cleanup, the

libraries were quantified fluorometrically using the Qubit™ dsDNA HS Assay (ThermoFisher, Waltham, MA, USA) and assessed for size distribution using a Fragment Analyzer (Advanced Analytical, Ames, IA, USA). Library concentrations were normalized, and the libraries were multiplexed with six libraries/pools.

Each multiplexed library pool was hybridized to a probe pool using the SeqCap EZ Human Exome Enrichment Kit v3.0 (Roche-NimbleGen, Madison, WI, USA). The enriched libraries were amplified with eight cycles of post-capture PCR, and assessed for exon target enrichment by qPCR. The exon-enriched libraries were then evaluated for size distribution using the Fragment Analyzer (Advanced Analytical) and quantified by qPCR using the KAPA Library Quantification Kit (KAPABiosystems). Sequencing was performed using a HiSeq4000 Sequencer (Illumina, San Diego, CA, USA.), one capture (six samples) per lane using the a 76 bp paired-end configuration.

WES data processing and genotyping quality check:

The raw output of the Illumina exome sequencing data was processed using Illumina's Consensus Assessment of Sequence and Variation (CASAVA) tool (v1.8.2) (http://support.illumina.com/sequencing/sequencing_software/casava.html) for demultiplexing and conversion to the FASTQ format. FASTQ files were aligned to the human reference genome (hg19) using BWA (v0.7.5) [11], allowing up to three mismatches (two mismatches must be in the first 40 seed regions) for a 76-base sequencing run. The aligned BAM files were then subjected to mark duplication, realignment, and base recalibration using Picard (v1.112) and GATK (v3.1-1) software tools [12]. The generated BAM files were then used

for downstream analyses. Genotyping quality check was performed to rule out any possible sample swapping or contamination. Briefly, germline SNPs were called using Platypus (v0.8.1) [13]. Samples from the same patient were confirmed and identified by the percentage of genotyping identity between them, which was defined as the fraction of identical germline alleles among the overlapping SNPs between the two samples. All samples in this study passed quality checks, and no sample swapping or contamination was detected.

Mutation calling, filtering, and functional annotation:

Somatic point mutations were identified using MuTect (v1.1.4) [14], and Pindel (v0.2.4) [15] was applied to identify small insertions and deletions (indels). The MuTect and Pindel outputs were then run through our pipeline for filtering and annotation. Only MuTect calls marked as "KEEP" were selected and used in the next step. For both substitutions and indels, mutations with a low variant allelic fraction (VAF < 0.02) or had a low total read coverage (< 20 reads for tumor samples; <10 reads for germline samples) were removed. In addition, indels that had an immediate repeat region within 25 base pairs downstream towards the 3' region were also removed. Common variants reported by the ExAc (the Exome Aggregation Consortium, http://exac.broadinstitute.org), Phase-3 1000 Genome Project (http://phase3browser.1000genomes.org/Homo_sapiens/Info/Index), or the NHLBI GO Exome Sequencing Project (ESP6500, http://evs.gs.washington.edu/EVS/) with a population minor allele frequency greater than 0.5% were removed. The intronic mutations, mutations at the 3' or 5' UTR or UTR flanking regions, silent mutations, small in-frame insertions, and deletions were also removed.

To evaluate the probability that a missense mutation is functionally deleterious, dbNSFP (v3.0) [16] was applied to add prediction scores for all missense mutations from 12 commonly used functional prediction algorithms: Polyphen-2 [17], SIFT [18], MutationTaster [19], Mutation Assessor [20], LRT [21], FATHMM-MKL [22] and DANN [23], PROVEAN[24], WEST3 [25], CADD [26], GERP++ [27], MetaSVM and MetaLR [28]. A missense mutation called "deleterious" by five or more algorithms was defined as a "deleterious" mutation.

Targeted Gene Panels

These gene assays, developed by our institution, were used in our patient cohort.

- End Lymphoma V1 – 162 targeted genes
- End CLL (Chronic Lymphocytic Leukemia) – 29 targeted genes
- End Leukemia – 81 targeted genes

Library Preparation:

Sequencing libraries were prepared from genomic DNA using hybridization capture-based target enrichment of the genomic regions of interest. Unique Molecular Indices (UMI) were included in the library preparation. A minimum family size of 2 was required per read to include sequencing data for further analysis. Bidirectional paired-end sequencing was performed using a next-generation sequencing (NGS) platform to screen for single nucleotide variants, and small insertions and deletions. The genomic reference sequence used was GRCh37/hg19. The software tools used were NextSeq Control Software 4.0.1, System Suite Version 4.0.1, and

Recipe Fragment Version 4.0.0. Sequence Analysis Viewer 2.4.5 IBM Spectrum LSF Application Center 10.2 BCL2FQ-CONCAT workflow 1.20 and SureCall, 4.1.2.11 Detailed information about signal processing, base-calling, alignment, and variant calling algorithms, are available upon request.

Analytical Sensitivity:

Tumor purity in the submitted sample was assessed by a combination of direct morphological assessment or immunophenotypic evaluation as part of the pathology workup. A minimum of 10% of the tumor cells is required in the sample for mutation analysis. For this assay, the detection sensitivity was partly related to the mutation depth of coverage, tumor percentage, and allelic frequency. Although the NGS platform is capable of achieving a much higher analytical sensitivity for clinical purposes, we determined that the effective lower limit of detection of this assay sensitivity was 5% (one mutant allele in the background of 19 wild-type alleles) by considering the depth of coverage of a given base and the ability to confirm low-level mutations using independent conventional platforms.

Variant Classification:

Variants identified from these panels are classified into three groups based on analytic findings, such as allelic frequency and currently available information in publicly available reference databases (COSMIC, dbSNP). Silent mutations, and prevalent germline polymorphisms (population frequency > 20%) in our clinical laboratory sample cohort are not reported.

Variants identified are:

- Probable somatic mutations
- Variants for which a germline versus somatic origin cannot be determined unequivocally.
- Variants reported as germline polymorphisms in population studies/literature/matched tumor-normal analyses of different patients in our laboratory.

In this study, we coded “probable somatic” and variants not equivocally somatic but leaning somatic as “somatic mutations.” Germline mutations were recorded per patient by gene name and were included as composite features in our study. Germline mutations suspected to be from clonal hematopoiesis of indeterminate potential identified (CHIP) from recent studies were included in a “clonal hematopoiesis” score which was determined from seven genes that were frequently mutated in CHIP (*IDH2*, *TET2, ASXL1, JAK2,* and germline *TP53* and *ATM* mutations*)*. The total number of germline variants was also recorded as we believe that some of these mutations are likely somatic in origin, but may or may not be pathogenic.

References

1. Younes A, Hilden P, Coiffier B, et al. International Working Group consensus response evaluation criteria in lymphoma (RECIL 2017). *Ann Oncol.* 2017;28(7):1436-1447.
2. Cheson BD, Fisher RI, Barrington SF, et al. Recommendations for initial evaluation, staging, and response assessment of Hodgkin and non-Hodgkin lymphoma: the Lugano classification. *J Clin Oncol.* 2014;32(27):3059-3068.
3. Kuhn M, Silge J. *Tidy Modeling with R.* " O'Reilly Media, Inc."; 2022.
4. Therneau T, Lumley T. R survival package. *R Core Team.* 2013.
5. *SHAPforxgboost: SHAP Plots for 'XGBoost'* [computer program]. Version 0.1.02020.
6. Greenwell BM, Boehmke BC, McCarthy AJ. A simple and effective model-based variable importance measure. *arXiv preprint arXiv:180504755.* 2018.
7. Schloerke B, Allen J. plumber: An API Generator for R package version 1.1. 0, 2021. *Reference Source.*
8. Jain P, Zhao S, Lee HJ, et al. Ibrutinib With Rituximab in First-Line Treatment of Older Patients With Mantle Cell Lymphoma. *Journal of Clinical Oncology.* 2021;40(2):202-212.
9. Wang ML, Jain P, Zhao S, et al. Ibrutinib–rituximab followed by R-HCVAD as frontline treatment for young patients (≤65 years) with mantle cell lymphoma (WINDOW-1): a single-arm, phase 2 trial. *The Lancet Oncology.* 2022;23(3):406-415.
10. Jain P, Zhang S, Kanagal-Shamanna R, et al. Genomic profiles and clinical outcomes of de novo blastoid/pleomorphic MCL are distinct from those of transformed MCL. *Blood Adv.* 2020;4(6):1038-1050.

11. Li H, Durbin R. Fast and accurate short read alignment with Burrows-Wheeler transform. *Bioinformatics.* 2009;25(14):1754-1760.

12. DePristo MA, Banks E, Poplin R, et al. A framework for variation discovery and genotyping using next-generation DNA sequencing data. *Nat Genet.* 2011;43(5):491-498.

13. Rimmer A, Phan H, Mathieson I, et al. Integrating mapping-, assembly- and haplotype-based approaches for calling variants in clinical sequencing applications. *Nat Genet.* 2014;46(8):912-918.

14. Cibulskis K, Lawrence MS, Carter SL, et al. Sensitive detection of somatic point mutations in impure and heterogeneous cancer samples. *Nat Biotechnol.* 2013;31(3):213-219.

15. Ye K, Schulz MH, Long Q, Apweiler R, Ning Z. Pindel: a pattern growth approach to detect break points of large deletions and medium sized insertions from paired-end short reads. *Bioinformatics.* 2009;25(21):2865-2871.

16. Liu X, Wu C, Li C, Boerwinkle E. dbNSFP v3.0: A One-Stop Database of Functional Predictions and Annotations for Human Nonsynonymous and Splice-Site SNVs. *Hum Mutat.* 2016;37(3):235-241.

17. Adzhubei I, Jordan DM, Sunyaev SR. Predicting functional effect of human missense mutations using PolyPhen-2. *Curr Protoc Hum Genet.* 2013;Chapter 7:Unit7 20.

18. Kumar P, Henikoff S, Ng PC. Predicting the effects of coding non-synonymous variants on protein function using the SIFT algorithm. *Nat Protoc.* 2009;4(7):1073-1081.

19. Schwarz JM, Cooper DN, Schuelke M, Seelow D. MutationTaster2: mutation prediction for the deep-sequencing age. *Nat Methods.* 2014;11(4):361-362.

20. Reva B, Antipin Y, Sander C. Predicting the functional impact of protein mutations: application to cancer genomics. *Nucleic Acids Res.* 2011;39(17):e118.

21. Chun S, Fay JC. Identification of deleterious mutations within three human genomes. *Genome Res.* 2009;19(9):1553-1561.

22. Shihab HA, Rogers MF, Gough J, et al. An integrative approach to predicting the functional effects of non-coding and coding sequence variation. *Bioinformatics.* 2015;31(10):1536-1543.

23. Quang D, Chen Y, Xie X. DANN: a deep learning approach for annotating the pathogenicity of genetic variants. *Bioinformatics.* 2015;31(5):761-763.

24. Choi Y, Sims GE, Murphy S, Miller JR, Chan AP. Predicting the functional effect of amino acid substitutions and indels. *PLoS One.* 2012;7(10):e46688.

25. Carter H, Douville C, Stenson PD, Cooper DN, Karchin R. Identifying Mendelian disease genes with the variant effect scoring tool. *BMC Genomics.* 2013;14 Suppl 3:S3.

26. Kircher M, Witten DM, Jain P, O'Roak BJ, Cooper GM, Shendure J. A general framework for estimating the relative pathogenicity of human genetic variants. *Nat Genet.* 2014;46(3):310-315.

27. Davydov EV, Goode DL, Sirota M, Cooper GM, Sidow A, Batzoglou S. Identifying a high fraction of the human genome to be under selective constraint using GERP++. *PLoS Comput Biol.* 2010;6(12):e1001025.

28. Dong C, Wei P, Jian X, et al. Comparison and integration of deleteriousness prediction methods for nonsynonymous SNVs in whole exome sequencing studies. *Hum Mol Genet.* 2015;24(8):2125-2137.
